# A transient apical extracellular matrix relays cytoskeletal patterns to shape permanent acellular ridges on the surface of adult *C*. *elegans*

**DOI:** 10.1371/journal.pgen.1010348

**Published:** 2022-08-12

**Authors:** Sophie S. Katz, Trevor J. Barker, Hannah M. Maul-Newby, Alessandro P. Sparacio, Ken C. Q. Nguyen, Chloe L. Maybrun, Alexandra Belfi, Jennifer D. Cohen, David H. Hall, Meera V. Sundaram, Alison R. Frand

**Affiliations:** 1 Department of Biological Chemistry, David Geffen School of Medicine, University of California, Los Angeles, California, United States of America; 2 Department of Genetics, University of Pennsylvania Perelman School of Medicine, Philadelphia, Pennsylvania, United States of America; 3 Department of Neuroscience, Albert Einstein College of Medicine, Bronx, New York, New York, United States of America; Queens College, CUNY, UNITED STATES

## Abstract

Epithelial cells secrete apical extracellular matrices to form protruding structures such as denticles, ridges, scales, or teeth. The mechanisms that shape these structures remain poorly understood. Here, we show how the actin cytoskeleton and a provisional matrix work together to sculpt acellular longitudinal alae ridges in the cuticle of adult *C*. *elegans*. Transient assembly of longitudinal actomyosin filaments in the underlying lateral epidermis accompanies deposition of the provisional matrix at the earliest stages of alae formation. Actin is required to pattern the provisional matrix into longitudinal bands that are initially offset from the pattern of longitudinal actin filaments. These bands appear ultrastructurally as alternating regions of adhesion and separation within laminated provisional matrix layers. The provisional matrix is required to establish these demarcated zones of adhesion and separation, which ultimately give rise to alae ridges and their intervening valleys, respectively. Provisional matrix proteins shape the alae ridges and valleys but are not present within the final structure. We propose a morphogenetic mechanism wherein cortical actin patterns are relayed to the laminated provisional matrix to set up distinct zones of matrix layer separation and accretion that shape a permanent and acellular matrix structure.

## Author summary

Animal surfaces are often decorated with intricately shaped structures such as denticles, ridges, or scales that are composed of extracellular matrix materials. We don’t understand how those extracellular materials get sculpted into appropriate shapes, but most models propose that cellular protrusions initiate the process. Here we investigated the formation of nematode adult alae, which are three racing stripe-like cuticle ridges that run along the left and right sides of the body. We found that alae development requires the actin cytoskeleton and a set of temporary matrix components, both of which organize into longitudinal stripes that presage the final structure. Using electron microscopy, we saw no evidence for cell membrane protrusion or folding that would explain a ridged matrix pattern. Instead, we observed that the first sign of alae formation is the appearance of four small separations between different layers of the temporary matrix. We propose that cytoskeletal patterns are relayed to the to the temporary matrix to trigger delamination and subsequent zonal differences in matrix accumulation that establish the alternating valley and ridge pattern of the alae.

## Introduction

Apical extracellular matrices (aECMs) line all epithelial surfaces in contact with the environment. These aECMs vary in composition, but typically contain a mix of proteins, carbohydrates and lipids that are organized into recognizable layers. Some aECMs are soft and gel-like, but others form more rigid structures with characteristic shapes, such as the hook-like denticles or cuticle ridges of insects and nematodes [1,2] or the scales on butterfly wings [3]. Examples of aECM in humans include mucin- and proteoglycan-rich linings within many tube lumens [4–6]; the tectorial membrane, a flexible aECM sheet that relays sound waves within the inner ear [7]; hair, an amalgamation of keratinized cells and extracellular macromolecules [8]; and tooth enamel, a composite of calcium phosphate minerals [9]. Mutations that affect component matrix proteins cause various disease phenotypes [4,6,7,10]. Despite the widespread functional and medical significance of the aECM, the cellular and molecular mechanisms that sculpt apical matrices are not well understood.

In some cases, the shape of an aECM structure is molded at least in part by the shape of the underlying epithelium at the time of matrix deposition. For example, denticles and taenidial ridges on insect cuticles originate as actin-based cellular protrusions that subsequently become coated with aECM [2,11,12]. The cellular protrusions eventually withdraw, leaving the rigid aECM structures in place. Differences in matrix composition affect not only denticle or taenidia shape but also the apical domain architecture within the underlying cells, suggesting mechanical connections among the aECM, apical junctions, and the actin cytoskeleton. However, these mechanical links between aECM and the cytoskeleton have yet to be fully elucidated. Furthermore, some complex aECM structures such as nematode alae are not obviously associated with cellular protrusions [1,13], raising the question of how such acellular structures are shaped.

**Fig 1:**
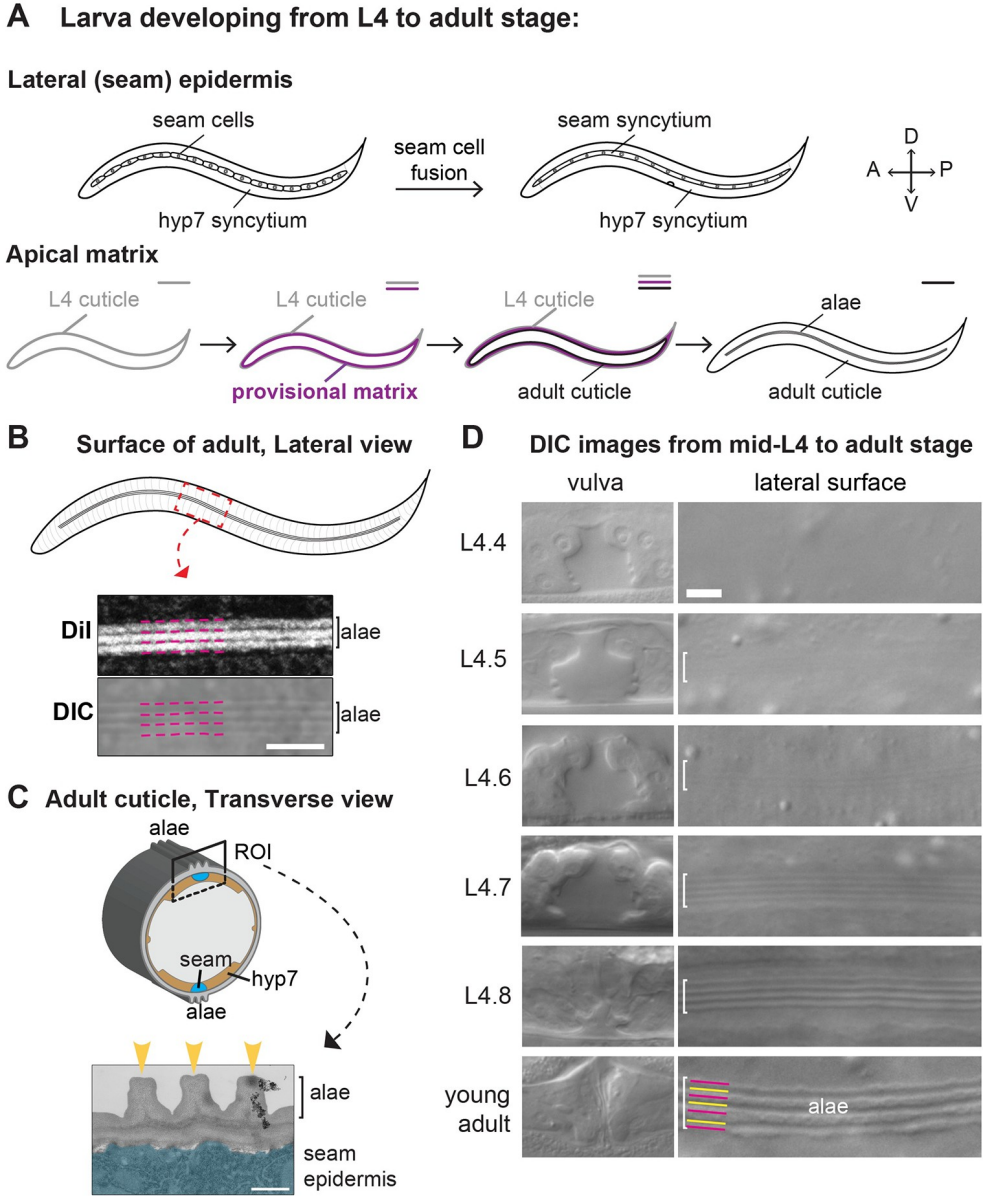
Timeline of adult alae formation. (A) L4-to-Adult development. Top schematics show anatomy of the seam and hyp7 syncytia. Bottom schematics show apical matrices. The provisional matrix (purple) is secreted beneath the L4 cuticle (gray) prior to, or during, synthesis of the new adult cuticle (black). (B) Lateral view of the adult cuticle. Micrographs show adult alae as visualized by DiI staining and DIC. Magenta lines indicate dark bands corresponding to valleys in both the DiI and DIC images. Scale bar: 5 μm. (C) Transverse view of adult. Cartoon (top) shows how alae are positioned relative to the seam and hyp7 syncytia. Transmission electron micrograph (bottom) shows the three alae ridges (yellow arrowheads). The underlying seam syncytium is false-colored in blue. Scale bar: 500 nm. (D) When viewed by DIC imaging, alae on the developing adult cuticle gradually became visible underneath the L4 cuticle. Brackets indicate regions above seam syncytia where longitudinal ridges could be detected. N2 animals were grown at 20°C and staged based on vulva tube morphology [31]. Alae were detected in 0/24 L4.4, 3/9 L4.5, 11/13 L4.6, 24/26 L4.7, 15/15 L4.8, and 16/16 adults imaged. When detected at L4.5 or L4.6, the alae were very subtle, as shown. Scale bars: 5 μm.

The *C*. *elegans* body cuticle is a multi-layered aECM composed mainly of collagens [14]. *C*. *elegans* sheds and replaces its cuticle by molting to progress between each of its four larval stages and to enter adulthood [15–17]. The cuticle of each stage is unique in structure: Longitudinal acellular ridges or "alae" form above the lateral (seam) epidermis in L1s, dauer larvae, and adults, but not in the intervening L2, L3, or L4 stages (Fig 1) [1]. Therefore, alae patterns must be generated *de novo*, rather than propagated from one life stage to the next across the molts. The number, size, and shape of alae ridges also varies among L1s, dauer larvae, and adults [1]. The basis of these stage-specific morphologies is unclear, except for the differential requirements for specific aECM proteins, including Zona Pellucida (ZP)-domain cuticulin proteins and various provisional matrix components [13,18–22].

Actomyosin-dependent forces have been indirectly implicated in alae formation at the L1 and dauer stages, but the specific steps involved have not been characterized in detail. L1 cuticle formation occurs in the context of actomyosin-driven seam cell apical constriction, which elongates the embryo and narrows the entire body shape [23,24]. Similarly, dauer larvae are radially-constricted compared to the preceding larval stage [16,19,22]. Cuticulin mutants that lack alae at one or both of these stages also have wider seam cells and a dumpy body shape [13]. Based on these observations, Sapio et al (2005) proposed that cross-linking of stage-specific ZP proteins shrinks the innermost cuticle layer to narrow the lateral epidermis and buckle the outer cuticle layers above it, forming the dauer alae. However, this "cuticle buckling" model does not readily explain how alae form in the adult, where overall volume increases while the general body shape remains similar to that of the preceding L4 stage (Fig 1A).

Recent work has shown that a ZP-rich provisional matrix precedes formation of each *C*. *elegans* cuticle [25]. In the embryo, the first cuticle is synthesized beneath a provisional matrix termed the embryonic sheath [23,26,27]. During the molt cycle, epidermal cells secrete a new provisional apical matrix beneath each pre-molt cuticle before synthesis of the post-molt cuticle (Fig 1A) [20,28]. Components of the provisional matrix are removed before or along with the pre-molt cuticle. This transient provisional matrix may help maintain tissue and body shape during the molting process. The provisional matrix also influences the organization of the subsequent cuticle and shapes the alae of the L1, dauer and adult stages, potentially by acting as a scaffold for further matrix deposition [18–20,28].

Here, we examined how the actin cytoskeleton and the provisional matrix work together to sculpt the alae of adult *C*. *elegans*. Transient actomyosin-dependent narrowing of the seam surface accompanies deposition of the provisional matrix at early stages of alae formation, but it does not appear to buckle the apical membrane. Instead, longitudinal actin filament bundles (AFBs) at the seam cortex align with ultrastructural delaminations and future valleys that flank alae ridges. Actin is required to pattern the provisional matrix into longitudinal bands, and the provisional matrix is required to establish demarcated zones of matrix layer adhesion and separation which ultimately give rise to alae ridges and their intervening valleys, respectively. We propose a morphogenetic mechanism wherein cortical actin patterns are relayed to the laminated provisional matrix to set up distinct zones of matrix layer separation and accretion, thereby shaping a permanent and acellular matrix structure.

## Results

### Morphogenesis of the adult-stage alae begins midway through the L4 stage

The *C*. *elegans* epidermis consists of cells and multinucleate syncytia that together synthesize most of the external cuticle [17,29]. The lateral seam and adjacent hyp7 syncytium are the two largest tissues and are connected by apical-lateral junctions analogous to those found in vertebrates and insects [30] (Fig 1A). The seam cells undergo stem cell-like asymmetric divisions early in each larval stage: anterior daughters fuse with hyp7, while posterior daughters remain in place and reconnect. During L4, seam cells exit the cell-cycle, fuse into bilateral syncytia, and ultimately synthesize alae—three key events that mark the L4-to-adult transition [17].

Adult alae consist of three longitudinal ridges that decorate the cuticle overlying the seam [1] (Fig 1). Alae ridges stain prominently with the lipophilic fluorescent dye DiI [32] (Fig 1B). Alae also can be seen with Differential Interference Contrast (DIC) microscopy as alternating dark and light stripes, which we confirmed correspond to the three ridges and four flanking valleys, respectively (Fig 1B). As viewed by transmission electron microscopy (TEM), alae are acellular structures and protrude approximately 0.5 microns above the rest of the cuticle surface (Fig 1C).

We were able to visualize the timing of adult alae formation using DIC microscopy of L4 animals staged based on developing vulva tube morphology (Fig 1D). Longitudinal stripes on the lateral surface first became visible at stages L4.5-L4.6. The stripes were initially subtle but gradually became more prominent at later stages. Thus, morphogenesis of the alae began well before the L4-to-adult molt and appeared to be a gradual rather than abrupt process.

### Actomyosin filaments in both the seam and hyp7 shape the adult-stage alae

To test the role of the actin cytoskeleton in forming alae, we used bacterial-mediated RNA-interference (RNAi) to silence actin genes in developing larvae and later examined the lateral surfaces of young adults by DIC microscopy and staining with DiI.

Five *C*. *elegans* genes encode actin monomers. During the L4 stage, epidermal cells and syncytia express *act-2* most highly, with some evidence for expression of *act-1*, *-3* and *-4* at other stages [33,34]. To simultaneously knock down *act-1*, *-2*, *-3* and *-4*, we selected an *act-2*-derived dsRNA trigger complementary to all four transcripts (Materials and Methods). Further, we customized and applied an established experimental paradigm to selectively knock down actin in either the seam or hyp7. This system involved tissue-specific expression of RDE-1, a worm homolog of Argonaute, in *rde-1(ne219)* mutants otherwise insensitive to siRNAs [35]. This approach bypassed much of the embryonic and larval lethality associated with systemic *actin(RNAi)* over the full course of development. Waiting until the L2 stage to deliver *actin* dsRNAs (an approach we term "attenuated RNAi") also bypassed much of this lethality, allowing larvae to develop into small adults.

**Fig 2:**
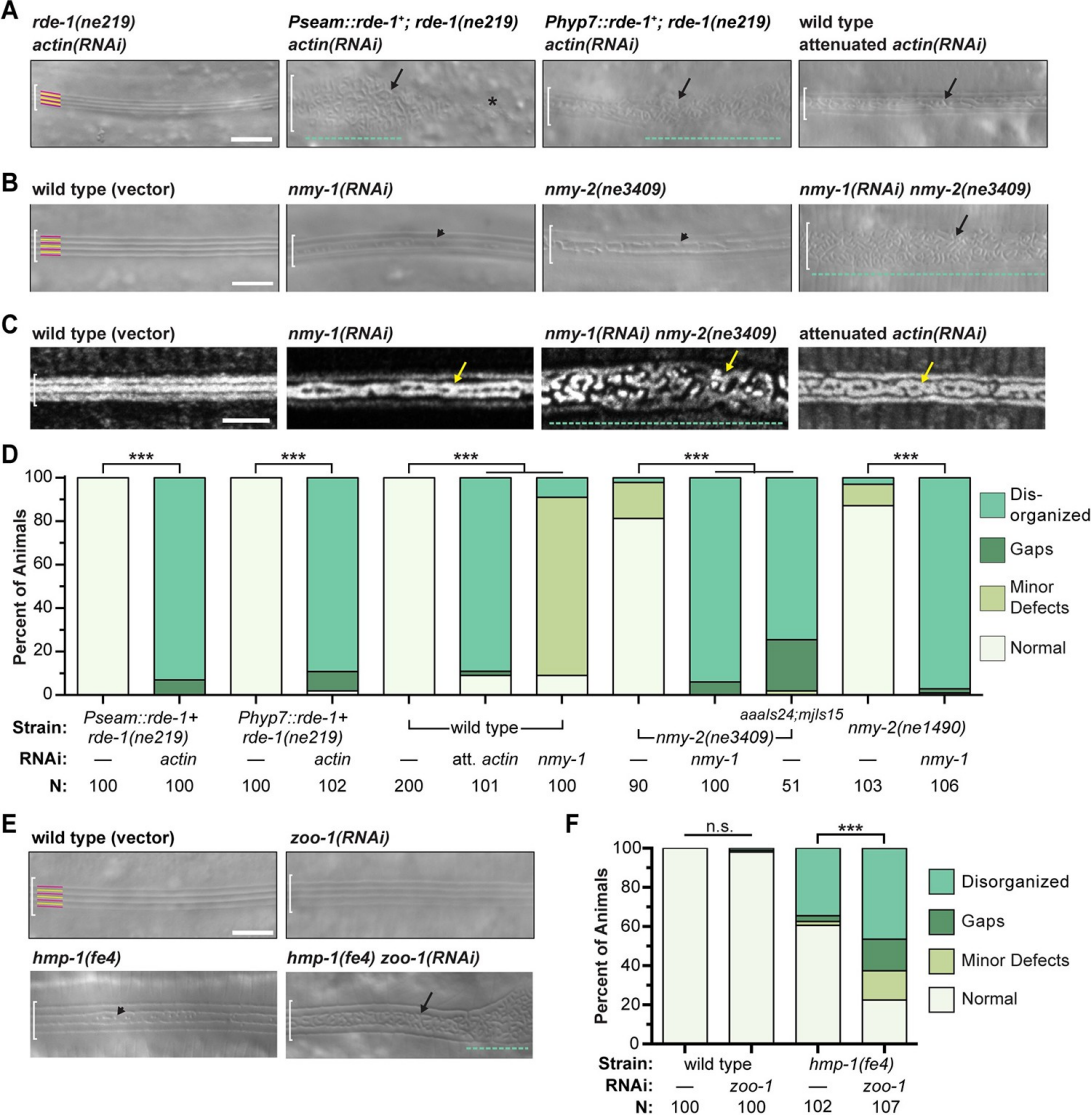
Knockdown of actin, NM II, or AJ components results in alae disorganization. (A) Tissue-specific and whole animal actin knockdowns (strains JK537, ARF281, ARF330, ARF408, N2, 25°C). *Pseam* is the promoter of *egl-18* (previously known as *elt-5*) [37]. *Phyp7* is the promoter of *dpy-7* [38]. (B) NM II mutants and knockdowns (strains WM179, WM180, 25°C). For both A and B, representative DIC images show structures on the lateral surface of young adults of indicated genotypes. White brackets demarcate the region of interest. Yellow and magenta lines label presumptive ridges and adjacent valleys in normal alae. Arrows point to tortuous sub-structures; arrowheads, minor deformities. Asterisk labels large gap. Green dashed lines label severely disorganized regions with no remaining longitudinal pattern. (C) DiI staining of the cuticle in actin and NM II knockdowns, with symbols as above. (D) Prevalence of deformed alae. Disorganized refers to alae that are fragmented and/or contain mis-oriented or tortuous ridges (arrows, green dashed lines). Gaps are regions >5 microns wide that lack alae entirely (asterisks). Minor deformities include smaller breaks and divots (arrowheads). Values are weighted averages from two independent trials; N: sample size. *** P<0.001 for all pairwise comparisons of prevalence of seemingly normal alae in knockdowns and mock-treated specimens; Fisher’s exact test with Bonferroni’s correction for multiple comparisons. (E-F) AJ mutants or knockdowns (strains N2 and PE97, 25°C), and prevalence of deformed alae, as above. ***P < 0.001. Scale bars: 5 μm.

Attenuated *actin(RNAi)* or preferential knockdown of actin in either the seam or hyp7 resulted in patches of disorganized adult-stage alae (Fig 2A and 2C and 2D). The most severely disorganized regions were widened and lacked continuous ridges entirely, instead showing a haphazard arrangement of short fragments. Other regions retained the outer dorsal and ventral ridges but showed breaks and misorientations within the central alae ridge, resulting in a “braid-like” appearance. Larger gaps in the alae (> 5 microns long) sometimes flanked the disorganized regions, and in these cases the underlying epidermis may have been disrupted due to earlier defects in seam cell reconnection, as previously described [36]. Because cell division and fusion were not of interest in this study, our approaches were designed to minimize such defects and our analysis prioritized extended regions of alae disorganization over any large gaps in the alae (Fig 2D, Materials and Methods). The abovementioned alae deformities were not observed in *rde-1* null mutants fed *actin* dsRNAs or in *wild-type* animals fed short dsRNAs transcribed from the vector. The fact that knockdowns in either the seam or hyp7 caused similar deformities suggests that actin networks within both syncytia work cooperatively to shape the adult-stage alae.

Non-muscle myosin (NM II) often partners with actin to generate morphogenetic forces [39,40]. The *nmy-1* and *nmy-2* genes of *C*. *elegans* both encode heavy chains of NM II that are 47% identical in primary sequence to human NMHC-IIB and expressed in the epidermis [33,41]. We used *nmy-1(RNAi)* and conditional alleles of *nmy-2* to partially inactivate NM II. Fragmented and disorganized alae were observed on most *nmy-1(RNAi); nmy-2(ts)* double mutants cultivated at restrictive temperature (Fig 2B–2D). In contrast, only minor deformities in the alae were observed in *nmy-1(RNAi)* or *nmy-2(ts)* single mutants, although *nmy-2(ts)* defects were greatly enhanced by expression of an F-actin biosensor and junction marker (see below) (Fig 2D). The combinatorial effect of *nmy-1(RNAi)* and *nmy-2(ts)* suggests that these paralogs make redundant contributions to a morphogenetic mechanism involving actomyosin-dependent forces.

### Apical Junction (AJ) components that interact with actin networks shape the adult alae

Actomyosin filaments often attach to cell membranes at cell-cell junctions [39,40]. To evaluate the role of AJs in patterning the adult alae, we similarly used RNAi and a hypomorphic allele to knock down key AJ components while larvae developed and then examined the lateral surface of young adults. HMP-1/α-catenin is the actin-binding component of cadherin-catenin complexes (CCCs) that mechanically link the various epidermal cells of *C*. *elegans* [30,42,43]. The Zonula Occludens (ZO) homolog ZOO-1 cooperatively recruits actin bundles to AJs [44]. Defective alae were observed on the surface of more than one third of surviving *hmp-1(fe4)* hypomorphic mutants, and these defects were further enhanced by simultaneous knockdown of *zoo-1* (Fig 2E and 2F). Thus, genetic manipulations known to impede the transmission of mechanical forces through AJs interfered with morphogenesis of adult-stage alae.

### Transient narrowing of the seam apical cortex precedes alae formation

**Fig 3:**
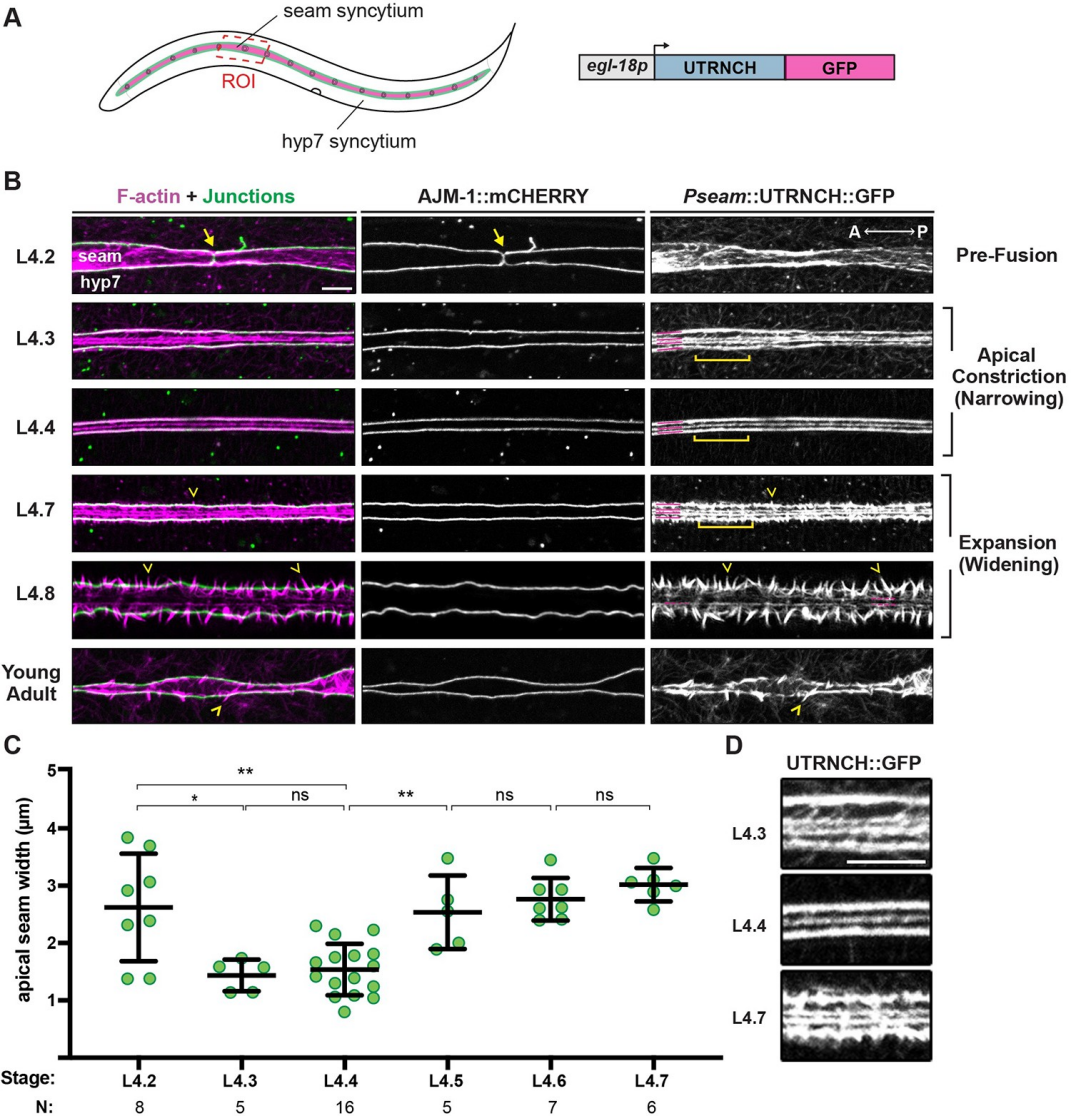
Formation of longitudinal AFBs and transient narrowing of seam syncytia precede the appearance of alae. (A) Diagram depicting seam (magenta) and hyp7 syncytia in an L4 larva. Green, apical junctions between syncytia. Red box indicates region of interest (ROI) imaged below. The seam-specific promoter *egl-18p* was used to drive UTRNCH::GFP expression. (B) Representative confocal projections show UTRNCH::GFP (magenta) and AJM-1::mCHERRY (green) signals captured at the indicated stages (strain ARF404, 25°C). Magenta lines label AFBs. Arrows point to AJs between seam (s) cell cousins about to fuse. Chevrons point to spikes of F-actin crossing AJs. Images are representative of at least n = 6 animals per stage, except n = 4 young adults. (C) Quantification of apical width of seam syncytia at indicated stages (strain SU93, 20°C). Values are the distance between AJM-1::GFP marked AJs, representing the mean of 6 measurements per worm. Bars: mean and s.d. *P ≤ 0.05, **P ≤ 0.01; Mann-Whitney test. (D) Enlarged ROIs delineated by brackets in (B). Scale bars: 5 μm.

To investigate the mechanism by which actomyosin networks pattern adult-stage alae, we further characterized the superficial shape of the seam epidermis and organization of cortical actin across the L4 stage. For this purpose, we used AJM-1 fusion proteins to label AJs among the seam and hyp7 syncytia (Figs 3A and 3B and S1) [45,46]. We also constructed and used a highly sensitive sensor for F-actin composing the Calponin homology domain (CH) of human Utrophin (UTRN) tagged with GFP and driven by the seam-specific promoter of *egl-18* [37] (Figs 3A and 3B and S1). This CH domain binds F-actin selectively and reversibly, and UTRNCH::GFP does not appreciably perturb actin dynamics when expressed at modest levels [47,48]. (Note however that we did observe genetic interactions between this actin sensor and several cytoskeletal or matrix mutants, see below). To achieve fine temporal resolution, we isolated precisely staged transgenic nematodes and imaged them at regular ~1hr intervals (Materials and Methods). We also collected images of the vulva to assess animal stage directly (Fig 1D).

The AJ marker revealed that seam cell fusion occurred immediately following the last round of cell divisions early in the L4 stage, with fusion complete before or during L4.2 (Figs 3B and S1). Soon after seam fusion, the apical surface of seam syncytia narrowed, appearing most narrow and uniform at L4.3 to L4.4 (Fig 3B and 3C). The apical surface of the seam then widened slowly, as the AJs spread apart over the following several hours and animals completed the L4/adult molt (Fig 3B and 3C). The observed cell shape changes suggested transient apical constriction on the dorsal-ventral (D-V) axis prior to initial alae formation, followed by gradual widening as the alae form and enlarge. The observation that alae form as the seam is widening seemed counter to the predictions of a membrane or matrix buckling model.

### Longitudinal AFBs in the seam presage the pattern of adult alae

The seam-specific UTRNCH::GFP marker revealed that striking changes in actin appearance accompanied seam cell division, fusion, narrowing, and widening (Figs 3B and 3D and S1). As seam cells reconnected following their last round of cell division, cortical actin networks remodeled to orient longitudinally. At the onset of narrowing (~L4.3), four longitudinal AFBs assembled at the cortex of the seam syncytia; two of these AFBs co-localized with AJM-1::mCHERRY along the dorsal and ventral junctions with hyp7, while the other two AFBs were located more medially. At the narrowest seam stage (L4.3-L4.4), often only three longitudinal AFBs were detected, suggesting the medial AFBs had moved closer together and potentially joined. As the seam widened again (L4.5-L4.7), four longitudinal AFBs were again observed.

Medial AFBs began to disassemble at the L4.7-L4.8 stages (Fig 3B), after the time that alae first become visible by DIC (Fig 1D). Breaks in the outer junctional AFBs also appeared at this time, and spikes of F-actin that apparently crossed the dorsal and ventral junctions became more prominent. This progressive transition from continuous longitudinal to discontinuous transverse F-actin structures along the margins might reflect a concurrent transition in the net direction of force propagation between the seam and hyp7.

In summary, the transient narrowing of seam syncytia midway through L4 coincided with dynamic reorganization of the bulk of cortical F-actin into four longitudinal AFBs. This pattern is not what we would expect for a typical apical constriction process, where AFBs are usually either isotropic or aligned in the direction of tissue narrowing [49]. However, these distinctive arrangements are reminiscent of the adult alae pattern of three longitudinal ridges and four flanking valleys, which first manifest while these AFB patterns are still present.

### Actin assembles into both longitudinal and circumferential AFBs in hyp7

**Fig 4:**
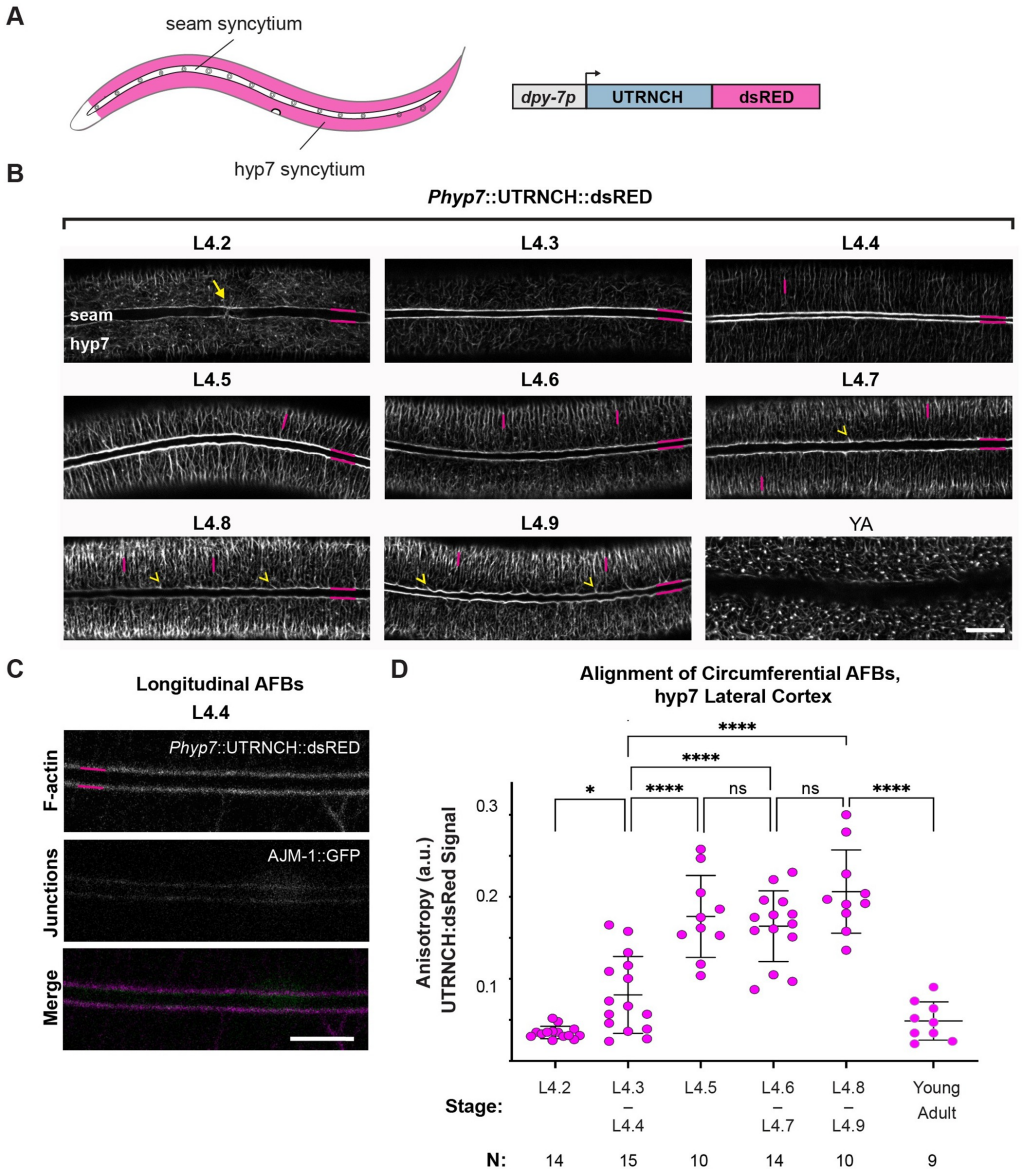
Longitudinal AFBs assemble in hyp7 as the seam narrows, whereas circumferential AFBs form parallel arrays as the seam widens. (A) Diagram as in Fig 3A except hyp7 is magenta while seam is white. The hyp7-specific promoter *dpy-7p* was used to drive UTRNCH::dsRED expression. (B) Inverted confocal fluorescence micrographs show UTRNCH::dsRED detected at the indicated stages (strain ARF385, 25°C). L4.2: Arrow indicates F-actin at a presumptive seam-seam junction before fusion. Horizontal magenta lines label longitudinal AFBs along the lateral margins. Vertical magenta lines label circumferential AFBs (CFBs). Images are representative of n = 9 or more animals per stage. Scale bar, 10 μm. (C) Longitudinal AFBs in hyp7 overlap with AJs. (D) Quantitation of cortical CFB alignment in lateral (thick) region of hyp7. Values are averages of 6 ROIs per worm. ****P ≤ 0.0001, *P ≤ 0.01, Anova with Tukey’s correction for multiple comparisons.

As our RNAi experiments indicated that actomyosin networks in hyp7 syncytia also contribute to morphogenesis of the alae (Fig 2), we went on to track the distribution of cortical actin in hyp7 across the L4 stage (Fig 4). For this purpose, we constructed a similar but distinct F-actin sensor comprising UTRNCH tagged with dsRED and driven by the hypodermal-specific promoter of *dpy-7* [38] (Fig 4A). This sensor revealed both longitudinal AFBs along the hyp7-seam margins and circumferential filament bundles (CFBs), some of which branched off from the longitudinal bundles (Fig 4B).

Like seam AFBs, the hyp7 longitudinal AFBs formed immediately following seam cell fusion (Fig 4B) and co-localized with an AJ marker (Fig 4C). These AFBs remained prominent throughout seam narrowing and widening (as defined in Fig 3) and then disappeared at the end of the L4-adult molt. We infer that longitudinal seam and hyp7 AFBs run in parallel along each side of the seam-hyp7 AJs.

The hyp7 CFBs have long been considered a hallmark of molting animals [26], but our analysis showed that they assemble earlier than previously thought, and that their appearance changes at the time of seam narrowing (Fig 4B and 4D). Prior to seam narrowing, some whisker-like filaments branched off from the longitudinal bundles at variable angles. Following seam narrowing, CFBs became increasingly anisotropic (aligned in parallel) up until the end of the L4-adult molt, when they collapsed at ecdysis (Fig 4B and 4D). Similar observations were recently reported by others [50] using a LifeAct reporter.

### Seam narrowing and AFB organization depend on NM II

**Fig 5:**
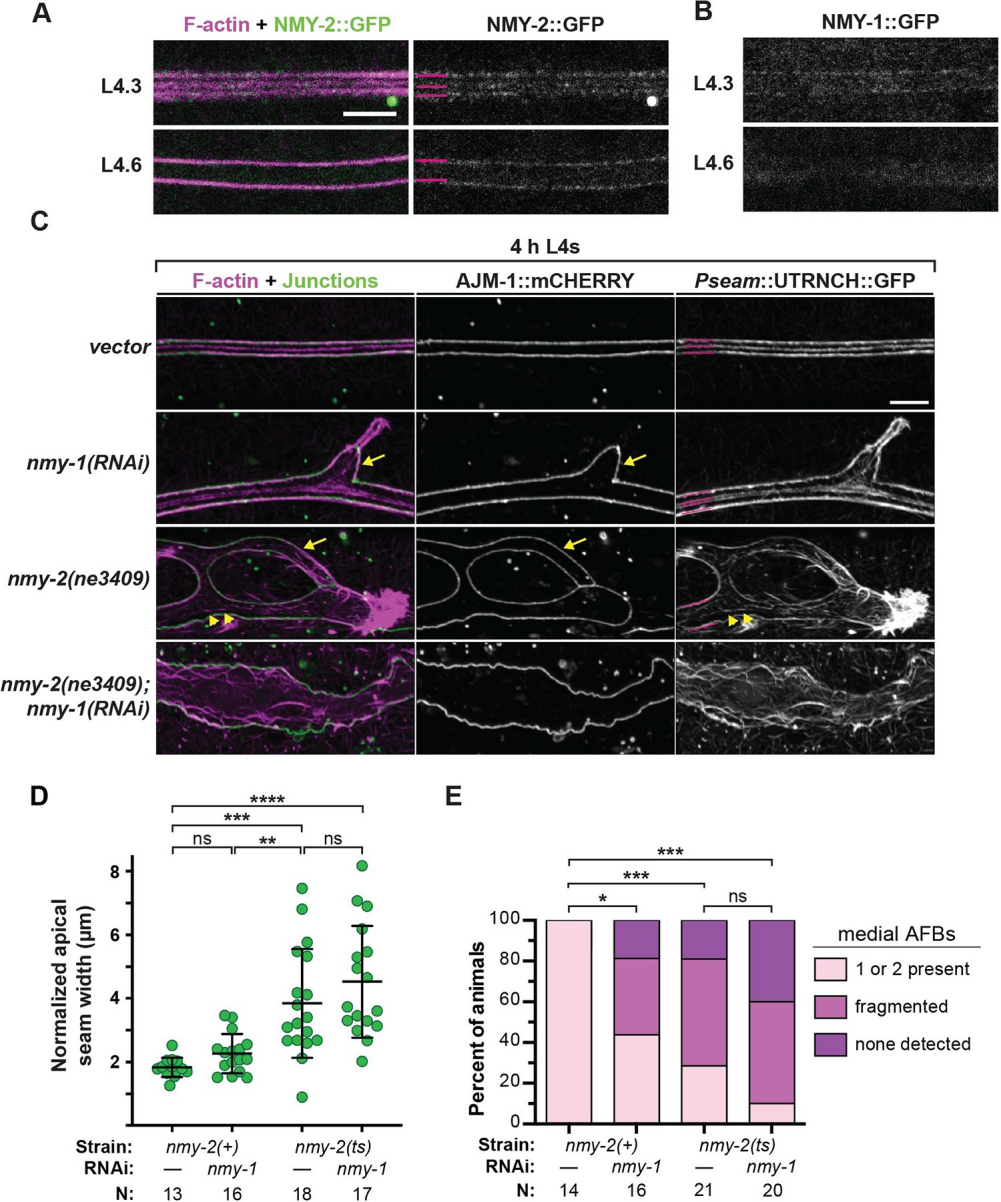
Longitudinal AFBs and contours of seam syncytia depend on NM II. (A) Endogenous NMY-2::GFP aligns with junctional AFBs at the seam-hyp7 margins and with the medial AFB in narrowed (L4.3-L4.4) seam syncytia. AFBs were marked by P*egl-18*::UTRNCH::dsRED (strain ARF500, 20°C). (B) Endogenous NMY-1::GFP also faintly marks seam-hyp7 margins (strain ML2540, 20°C). Both A and B show single confocal slices representative of at least 6 animals per stage. (C) Representative confocal maximum projections show F-actin and AJs in seam syncytia of mid-L4 animals that expressed *Pegl-18*::UTRNCH::GFP and AJM-1::mCHERRY (strain ARF404, 25°C). Magenta lines label longitudinal AFBs. Arrows point to extensions of seam over hyp7. Asterisks label aggregated F-actin. Arrowheads point to fragmented medial AFBs. Scale bar: 5 μm. (D) Quantitation of seam width—values are the area surrounded by AJs normalized to imaged interval. Bars signify mean and sd; ****P ≤ 0.0001, ***P ≤ 0.001, ** P ≤ 0.01, ordinary Anova with Tukey’s test for multiple comparisons. Images and measurements from two independent trials; total sample sizes as indicated.(E) Quantitation of UTRNCH::GFP patterns. *P <0.01, ***P <0.0001, Fisher’s Exact test.

If AFBs and/or CFBs are contractile structures, we would expect them to associate with NM II [39,40]. To test this, we examined the localization patterns of NMY-1::GFP and NMY-2::GFP expressed from the endogenous loci [27,51], and we generated a seam UTRNCH::dsRed reporter and compared the patterns of actin and NMY-2::GFP (Fig 5A and 5B). Surprisingly, while UTRNCH::dsRed marked three longitudinal AFBs upon seam narrowing, it differed from UTRNCH::GFP in that, during widening, it clearly marked only the two junctional AFBs and only faintly if at all marked the two medial AFBs (Fig 5A). One difference between GFP and dsRed is that the latter is an obligate tetramer [52], so differences in conformation might explain the discrepancy in the patterns seen with these two actin sensors. Whatever the explanation, the differential ability of UTRNCH::dsRed to mark junctional vs. medial AFBs suggests that these AFBs may differ in organization.

NMY-1::GFP and NMY-2::GFP puncta aligned along or near the junctional AFBs at the hyp7- seam margins, both during and following seam narrowing (Fig 5A and 5B). NMY-2 puncta also marked the transient medial AFB(s) at the narrowest seam stage but did not appear to mark medial AFBs at later stages (Fig 5A). Neither NMY-1 nor NMY-2 detectably marked hyp7 CFBs at the mid-L4 stages examined (n = at least 12 each), although we can’t exclude low levels below our detection. These data suggest that, at the time of seam narrowing, the longitudinal AFBs are part of a contractile actomyosin network. However, during seam widening and alae formation, the medial longitudinal AFBs (visible with UTRNCH::GFP) may no longer be contractile.

To test if NM II-dependent actomyosin constriction drives the observed changes in seam syncytium shape, we examined this tissue in NM II knockdowns at L4.4 (Fig 5). Both *nmy-1(RNAi)* and *nmy-2(ts)* single mutants displayed misshapen syncytia with ectopic branches and protrusions (Fig 5C). *nmy-1(RNAi)* larvae had seam widths only slightly larger than those of age-matched controls, while *nmy-2(ts)* single mutants (which show genetic interactions with the actin sensor—Fig 2D) or *nmy-2(ts); nmy-1(RNAi)* double mutants had significantly distended seam syncytia, on average more than twice as wide as controls (Fig 5C and 5D). Therefore, seam narrowing and overall seam shape depend on NM II.

To further characterize the relationship between NM II and the various actin structures observed, we examined the distribution of UTRNCH::GFP in NM II knockdowns. Junctional AFBs were variably detected in *nmy-1(RNAi) nmy-2(ts)* double mutants and medial AFBs were largely fragmented or absent (Fig 5C and 5E). These findings suggest that NM II is required for proper assembly or maintenance of seam actin networks and are consistent with the model that these actin networks pattern the eventual alae.

### Provisional matrix components are required for adult alae shaping but not AFB assembly

**Fig 6:**
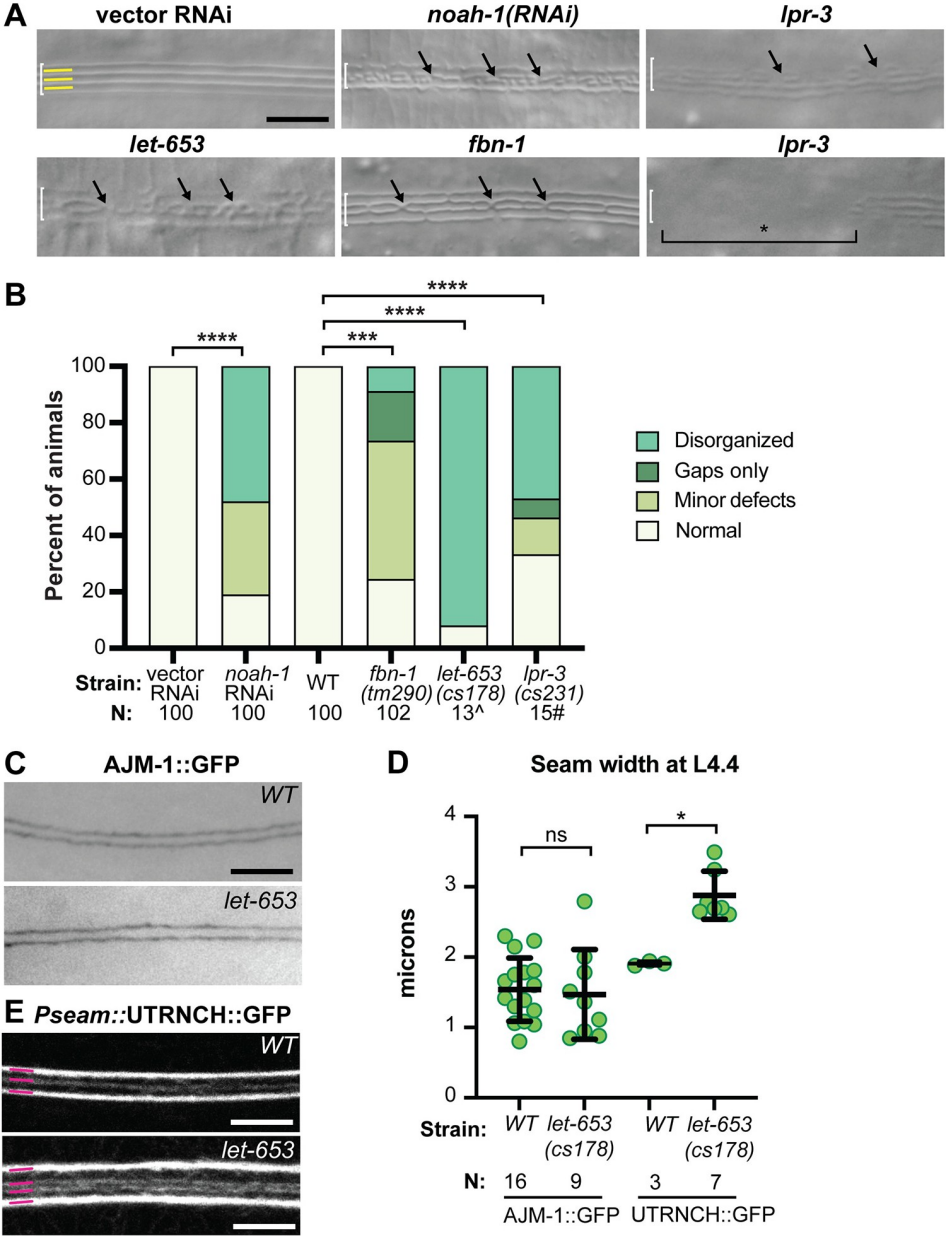
Provisional matrix components are required for patterning the alae. (A) DIC images of alae in RNAi knockdowns or mutants of the indicated genotypes (Strains N2 and ARF251, 25°C; strains UP3184 and UP3452, 20°C). Arrows indicate disorganized regions. Bracket indicates a large gap where no alae are present. (B) Quantification of alae defects, as in Fig 2D. ****P<0.0001, Fisher’s exact test. ^Data reproduced from [20]. ^#^Numbers here are an under-estimate of the true penetrance of alae defects because not all mosaics would have lost *lpr-3* in the seam lineage (see Materials and Methods). (C) At L4.4 stage, seam width (visualized with AJM-1::GFP) is similar between *WT* (strain SU93) and *let-653(cs178)* mutants (strain UP3184), both at 20°C. (D) Measurements were performed as in Fig 3B, using either AJM-1::GFP or the actin sensor UTRNCH::GFP to assess seam margins. *WT* datapoints for AJM-1::GFP are re-used from Fig 3B. *let-653* genetically interacted with the actin sensor to widen the seam. ns = not significant. *P<0.05, Mann-Whitney test. (E) At L4.4 stage, longitudinal AFBs (visualized with Pseam::UTRNCH::GFP) were present in both *WT* (strain ARF505) and *let-653(cs178)* mutants (strain UP4195), both at 20°C.

The misshapen or fragmented adult alae seen after actin, NM II, or junctional cadherin depletion are similar to those previously reported in some mutants affecting the provisional matrix that precedes each cuticle [20,28]. To test if other known components of this matrix are also required to pattern the adult alae, we used RNAi knockdown (*noah-1*), mutant escapers (*fbn-1*), or mosaic approaches (*lpr-3*) to circumvent the early arrest phenotypes seen in null mutants. RNAi knockdown or loss of the ZP proteins NOAH-1 or FBN-1 resulted in alae deformations similar to those previously reported after loss of the ZP protein LET-653 [20] (Fig 6A and 6B). Mosaic animals losing the lipocalin LPR-3 in subsets of seam cells (see Materials and Methods) also had regions of misshapen or fragmented alae and, in some cases, regions where alae were entirely missing (Fig 6A and 6B). Therefore, the provisional matrix appears broadly important for forming and shaping adult alae.

Sapio et al (2005) proposed that, in L1 and dauer larvae, the ZP matrix drives seam narrowing. To test if ZP proteins in the provisional matrix are required for seam narrowing during the L4 stage, we examined *let-653* mutant L4s obtained by rescuing the lethal embryonic excretory tube defects with a tissue-specific rescue transgene (Materials and Methods). These mutants have defective alae (Fig 6A and 6B) but are otherwise healthy. Seam morphology in these animals was examined with AJM-1::GFP. At L4.4, *let-653* mutants had fully fused and narrow seam cells that resembled those in wild-type animals (Fig 6C and 6D), so at least this matrix component is not required for seam narrowing. Consistent with this, *let-653* mutants still assembled longitudinal AFBs (Fig 6E). However, *let-653* did genetically interact with our actin sensor, *Pseam*::UTRNCH::GFP, to compromise seam narrowing (Fig 6D and 6E), suggesting cross-talk between the cytoskeleton and the provisional matrix.

### Provisional matrices are patterned into longitudinal bands during seam widening

**Fig 7:**
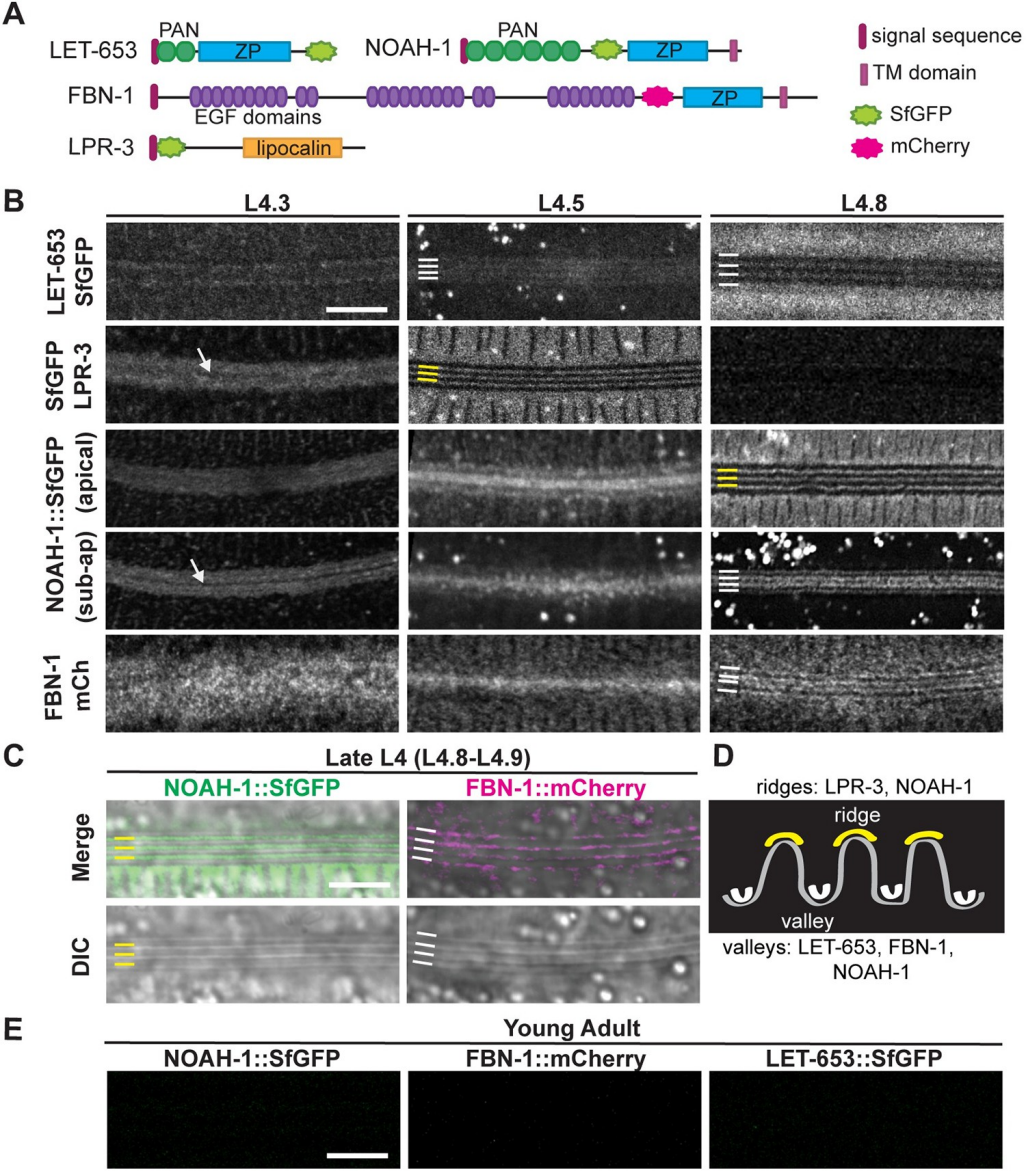
Provisional matrix patterns presage the ridges and valleys of adult-stage alae. (A) Diagrams of provisional matrix components. (B) Representative confocal slices show the dynamic distributions of indicated fusions in animals from mid- to late-L4. LET-653::SfGFP (strain UP3746, 20°C). SfGFP::LPR-3 (strains UP3666 or UP3693, 20°C). NOAH-1::SfGFP (strain ARF503 25°C). FBN-1::mCHERRY (strain ARF379, 25°C). For NOAH-1::SfGFP, both apical and sub-apical confocal slices from the same animal are shown; 1/8 and 3/8 late L4 animals showed only the "ridge" or only the "valley" pattern, respectively, and 4/8 showed both patterns simultaneously. (C) Airyscan-processed images of late L4s, showing that apical NOAH-1 aligns with alae ridges and sub-apical FBN-1 aligns with valleys, as seen by DIC. (D) Schematic shows interpretation of apical patterns as alae ridges and sub-apical patterns as valleys. Correspondingly, on all micrographs, yellow lines indicate developing alae ridges and white lines indicate flanking valleys. (E) Provisional matrix factors disappear in adults. Confocal slices from same strains shown in A, at 20°C 24 hours after mid-L4 stage. All images are representative of at least n = 5 per marker per stage. Scale bars: 5 μm.

To examine the timing and patterns of provisional matrix deposition, we visualized the lipocalin LPR-3 and the ZP-domain proteins LET-653, FBN-1, and NOAH-1 using functional translational fusions expressed from the endogenous loci or from extrachromosomal transgenes [27,53] (Materials and Methods) (Fig 7A). Provisional matrix proteins were visible over the seam syncytium during the L4.3-L4.4 stages, suggesting they are secreted prior to and/or during the period of seam narrowing (Fig 7B). The provisional matrix proteins initially appeared diffuse and unpatterned, but over the next few hours, each protein resolved into a characteristic pattern of longitudinal bands that correspond to developing alae ridges or their associated valleys and borders (Fig 7B–7D). LPR-3 and LET-653 bands appeared in mid-L4, before alae became clearly detectable by DIC. LPR-3 specifically marked three developing ridges, while LET-653 marked four valleys (Fig 7B). NOAH-1 bands resolved slightly later and marked both three ridges and four valleys in different z-planes (Fig 7B–7D). The distance between the NOAH-1 apical ridge signal and the subapical "valley" signal was 0.9 microns, which is greater than the height of the mature alae; from this we infer that subapical NOAH-1 may sit at the plasma membrane below the new adult cuticle. FBN-1 was the last factor to become patterned, very close to the L4-Adult molt, and marked only valleys (Fig 7B–7D). Each protein also showed a different timeline of disappearance, but all disappeared from the alae region in adults (Fig 7E). Therefore, these provisional matrix proteins are present during the period when alae are first being patterned and formed, but they are not permanent components of these ridge structures.

### Actin is required to pattern the provisional matrix into longitudinal bands

**Fig 8:**
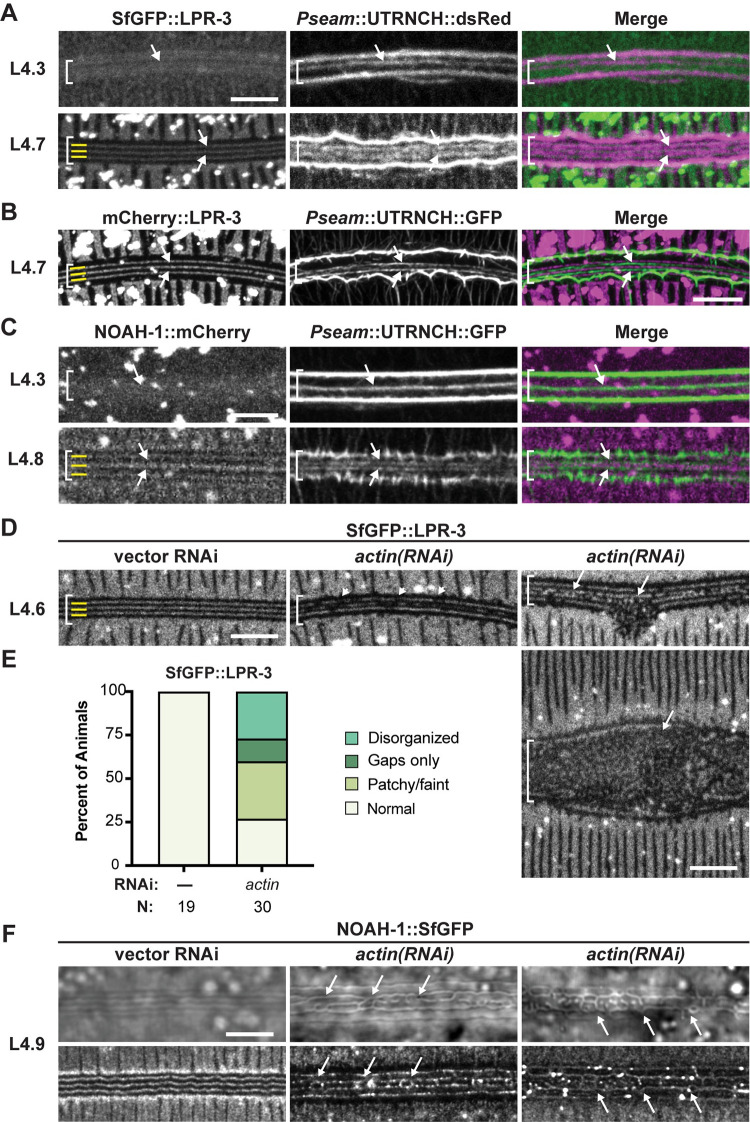
Actin is required to pattern the provisional matrix. (A-C) Spatial relationships between seam AFBs and provisional matrix bands. Maximum intensity projections of animals expressing the designated actin sensor and matrix fusion. Brackets indicate seam region. Arrows point to medial AFBs. Images are representative of at least n = 6 per strain per stage. (A,B) LPR-3 apical bands are largely offset from AFBs (strains UP4127 and UP4170, 20°C). For A, only specimens where the UTRNCH::dsRed sensor detected medial AFBs in addition to junctional AFBs could be assessed. (C) NOAH-1::SfGFP apical bands are largely offset from AFBs (strain UP4114, 20°C). D) *actin RNAi* disrupts LPR-3 provisional matrix patterns. Standard methods for bacterially-induced *actin RNAi* were used, and surviving animals were imaged at the L4.5-L4.7 stage (strain UP3666, 20°C). Middle panel shows example of a patchy and faint pattern. Right panels show examples of disorganized patterns. (E) Quantitation of SfGFP::LPR-3 patterns after actin depletion. (F) *actin RNAi* disrupts NOAH-1 provisional matrix patterns. The attenuated *actin RNAi* protocol was used, and animals were imaged at the late L4 stage (Strain ARF503, 25°C). 5/8 specimens showed alae abnormalities that matched the aberrant NOAH-1 pattern, while 3/8 had normal alae and normal NOAH-1 bands. Scale bars: 5 μm.

To understand the relationships between the seam longitudinal AFBs and the overlying longitudinal bands of provisional matrix, we combined our seam actin sensors with different matrix fusions (Fig 8A–8C). In narrowed seams, when only one medial AFB could be resolved, SfGFP::LPR-3 was cleared from a thin band directly overlying that medial AFB (Fig 8A). A similar largely offset relationship was observed between the AFBs and the 3 apical bands of LPR-3 or NOAH-1 in later L4 animals (Fig 8A–8C). Together, these data suggest that AFBs underlie nascent valleys surrounding each developing ridge.

To test if AFBs might pattern the provisional matrix, we determined the effect of actin RNAi on provisional matrix patterns. Following actin knockdown, SfGFP::LPR-3 often appeared greatly disorganized or localized to misoriented or braid-like structures, rather than longitudinal bands (Fig 8D and 8E). Furthermore, the remaining SfGFP::LPR-3 bands frequently contained many small breaks and regions that were faint and ill-defined (Fig 8D and 8E). Similarly, in older larvae in which disorganized alae ridges were becoming detectable by DIC, NOAH-1::SfGFP marked the misoriented ridges (Fig 8F). Finally, in L3 larvae where cortical actin networks are more isotropic, both LPR-3 and NOAH-1 were present but remained diffuse and unpatterned over the seam (S1 Fig). We conclude that the L4-specific cortical actin networks are required to pattern the provisional matrix into continuous longitudinal bands to initiate alae formation, and that loss or mis-patterning of the provisional matrix can explain mis-patterning of the permanent alae ridges.

### Ultrastructure of developing alae reveals alternating sites of separation and adhesion among apical matrix layers

**Fig 9:**
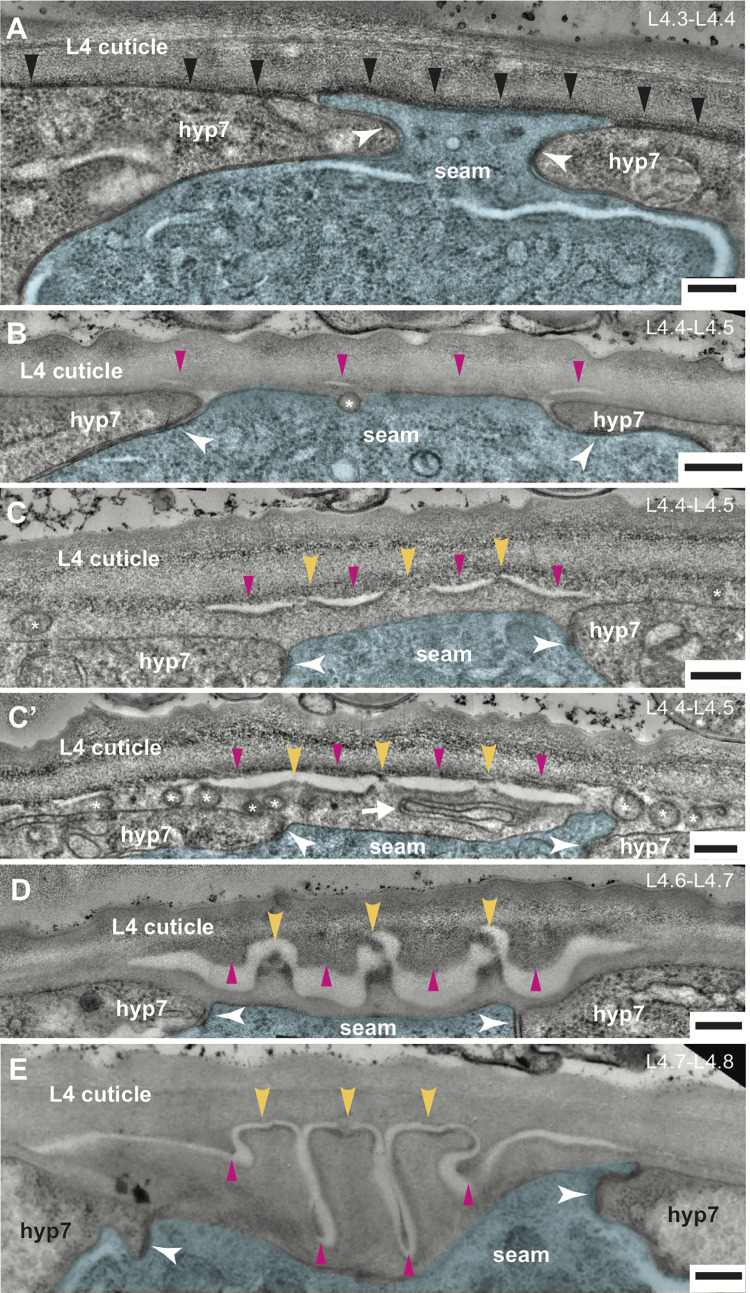
Ultrastructure of developing alae reveals differential matrix separation vs. adhesion. (A-F) TEM micrographs of mid- to late-L4 *wild-type* (N2) specimens arranged by inferred age (total N = 10). Transverse cuts through the mid-body are shown. See Fig 1C for cartoon rendering of perspective. Seam cell is false-colored in blue. White arrowheads indicate adherens junctions between the seam and hyp7 syncytia. Scale bars: 200 nm. (A) ~L4.3-L4.4. The seam cell is highly constricted, with its narrowest point ~500nm in width. Black arrowheads indicate electron dense provisional matrix material on apical surfaces of both seam and hyp7 syncytia. (B) ~L4.4-L4.5. Magenta arrowheads indicate four regions of provisional matrix separation. Asterisk marks a vesicle in transit across seam membrane. (C) ~L4.4-L4.5. Yellow arrowheads indicate three regions of provisional matrix adhesion at nascent alae tips. Extracellular vesicles (asterisks) are present in the matrix over hyp7. C’) Regions of matrix separation are enlarged compared to panel C, which is another body region from the same specimen. Many extracellular vesicles (asterisks) and a larger membrane-bound structure (arrow) are present within the future adult cuticle. (D) ~L4.6-L4.7. Discernable alae ridges have formed and contain electron dense material at their tips. Matrix fibrils connect these ridges to the L4 cuticle, while additional L4-cuticle-attached matrix protrudes down into the intervening gaps. (E) ~L4.8. Maturing alae have grown in length and width, and valleys have narrowed. The central ridge still maintains a discernable connection to the L4 cuticle.

To characterize changes in the ultrastructure of the lateral epidermis and overlying apical matrices that happen while the alae take shape, we turned to transmission electron microscopy (TEM), using high pressure fixation to best preserve the fragile matrix [54,55]. We collected transverse sections through the mid-body of 10 distinct mid-L4 specimens, inspected the corresponding micrographs, and ordered the specimens by inference based on matrix appearance and comparisons to our DIC (Fig 1D) and confocal (Figs 3 and 4 and 7) image timelines. The micrographs shown in Fig 9 represent distinct steps in alae morphogenesis.

In two inferred L4.3-L4.4 stage specimens (Fig 9A), the subapical region of the seam cell (at the adherens junctions) appears very narrow and pinched (0.5–1 micron wide), with hyp7 pushing in on both sides, consistent with apical constriction. Dark electron-dense extracellular material sits beneath the L4 cuticle, lining the apical plasma membrane of both the seam cell and hyp7; this material likely corresponds to the provisional matrix, which is deposited at this stage (Figs 7 and 8). The seam cell apical membrane and L4 cuticle remain flat in these specimens. However, in a third specimen that also appears to be ~L4.4, the seam cell apical surfaces are still flat, yet the L4 cuticle has seemingly buckled into three deep folds (S2 Fig). This latter animal has a large break in the L4 cuticle at the vulva lumen, which may have released mechanical constraints on the tissue and matrix. Alternatively, cuticle buckling could be a normal but transient response to initial seam constriction.

In three specimens inferred to be just slightly older, about L4.4 to L4.5 stage (Fig 9B–9C’), the seam apical surface is narrow (0.9–1.1 microns) and the apical membrane and L4 cuticle are flat, but four discrete regions of separation appear between matrix layers over the seam. These separations define three intervening regions of remaining matrix adhesion, which we infer correspond to the future alae ridges. When comparing two images from different body regions in one of these specimens (Fig 9C–9C’), the separations are larger, and points of adhesion narrower, as ridges become more apparent. Dark electron-dense material lines the top and bottom surfaces of the separations, suggesting that both separations and adhesions occur between layers composed of the freshly deposited provisional matrix.

Another feature of these three specimens is the presence of many membrane-bound vesicles or organelles near the seam and hyp7 apical membranes, including in the apparent extracellular space where new adult cuticle is forming (Fig 9B–9C’). The extracellular vesicles (EVs) range in size from ~15 nm (the typical size of exosomes [56]) to >600 nm (resembling migrasomes [57]). These EVs may contain materials for building or modifying the alae and cuticle.

**Fig 10:**
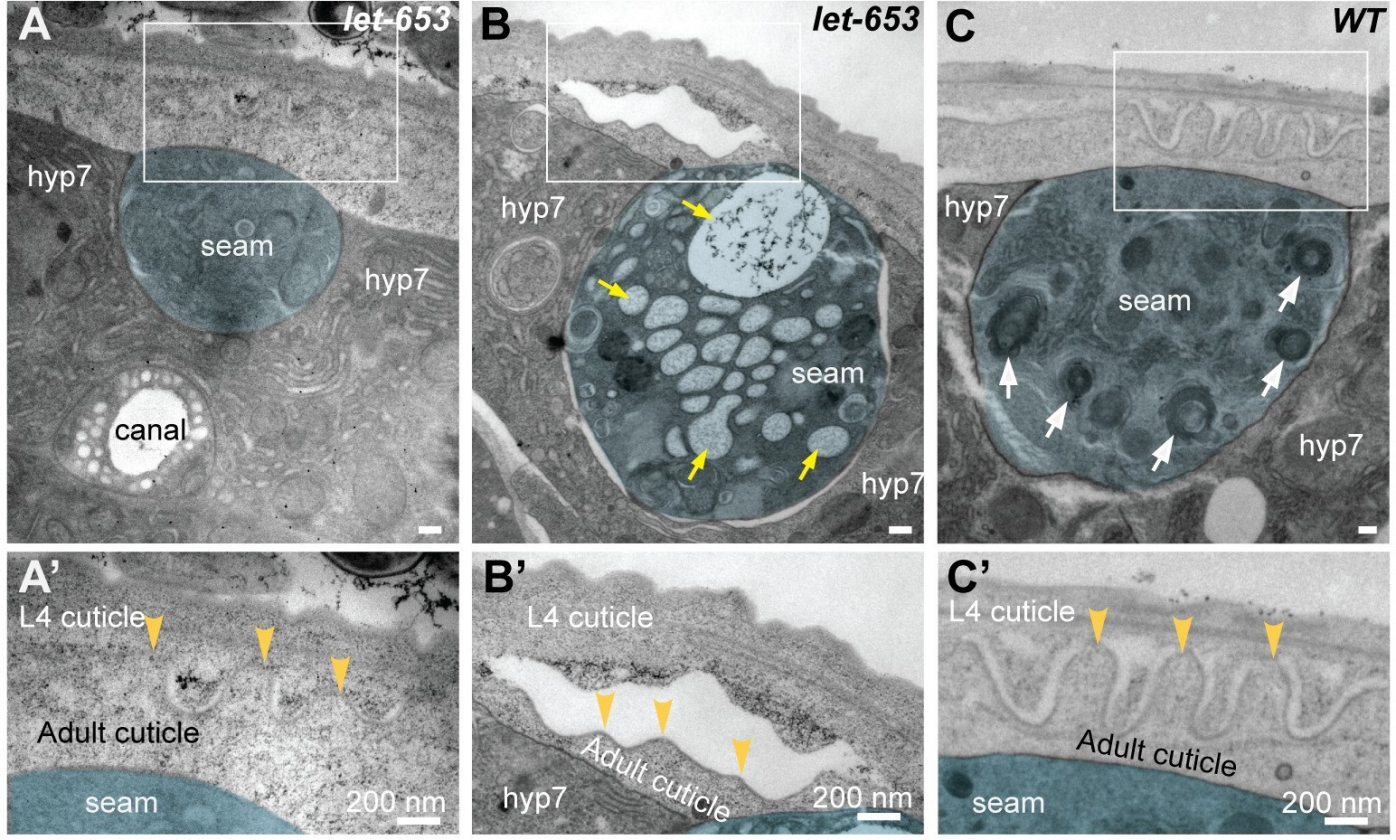
The provisional matrix component LET-653 is required for patterned adhesion versus separation of matrix layers. (A-C) TEM micrographs of *let-653(cs178)* (A,B, strain UP3342) or *wild-type* (C, N2) L4 specimens. Transverse cuts through the mid-body are shown. Seam cell is false-colored in blue. Scale bars: 200 nm. Boxed regions are shown at higher magnification in A’-C’. (A, A’) There is no clear distinction of adhesive *vs*. separated matrix regions in this mid-L4 *let-653* specimen, despite the appearance of nascent alae ridges. (B, B’) Mis-shapen alae ridges have completely separated from the L4 cuticle of this late L4 *let-653* specimen. Large vesicle structures (yellow arrows) also fill the seam cell, suggesting abnormal protein trafficking, We previously reported an accumulation of unusually large vesicles in vulF vulva cells of this same specimen [53]. (C, C’) Many lysosomes or related lamellar organelles (white arrows) appear within the seam cell in wild-type late L4 specimens (N = 4).

In the four oldest specimens, inferred to range from L4.6 to L4.8, the seam cell is wider (1.5–3 microns) and in three of the specimens it has sunk internally, below the level of hyp7 (Fig 9D and 9E). The seam cell contains many lamellar structures resembling lysosomes or lysosome-related organelles (LROs) (Fig 10C). The nascent adult cuticle underneath the L4 cuticle shows progressively larger alae ridges, with deeper and narrower valleys separating the ridges. Small points of connection remain between these adult alae ridges and the thinning L4 cuticle above. Much additional matrix material has accreted at the base of the L4 cuticle and protrudes downward in a pattern that is complementary to that of the adult alae. This material is presumably "valley-localized" provisional matrix (Fig 7C and 7D) that will be removed along with the old L4 cuticle at the molt.

From these TEM data, we draw several key conclusions. First, many changes in matrix appearance occur immediately following the apex of seam narrowing. These changes are accompanied by the presence of vesicle populations, including EVs, that suggest active secretion by both the seam and hyp7 syncytia. Second, the seam apical membrane appears smooth throughout all stages of alae formation, with no evidence of upward protrusions or folds. However, the seam does sink internally as alae form, suggesting some downward force. Third, formation of alae involves differential separation vs. adhesion of matrix layers within the provisional matrix zone, with four regions of separation and three intervening regions of adhesion echoing the spacing of longitudinal AFBs within the widening seam and the banding patterns observed for provisional matrix proteins.

### Ultrastructure of the seam aECM in *let-653* mutants reveals requirements for the provisional matrix in patterning differential matrix adhesion

The above TEM data suggested that differential matrix separation occurs between layers of the provisional matrix. To better understand the contribution of provisional matrix to alae patterning, we also examined *let-653* mutant ultrastructure by TEM.

Fig 10 shows transverse TEM sections through the lateral epidermis of two mid-L4 *let-653* larvae processed identically and at the same time as the wild-type specimens above. In the first specimen, which appears to be the youngest of the two, developing alae ridges are discernible above the seam cell, but there are no clear distinctions between regions of matrix separation and adhesion (Fig 10A). In the second specimen, the nascent alae cuticle has separated entirely from the above L4 cuticle (Fig 10B). These data show that *let-653*, and likely the entire provisional matrix, is required for proper patterning of matrix separation vs. adhesion during alae formation. Thus, while some ridges do form in the mutants, they are not properly shaped or continuous.

## Discussion

**Fig 11:**
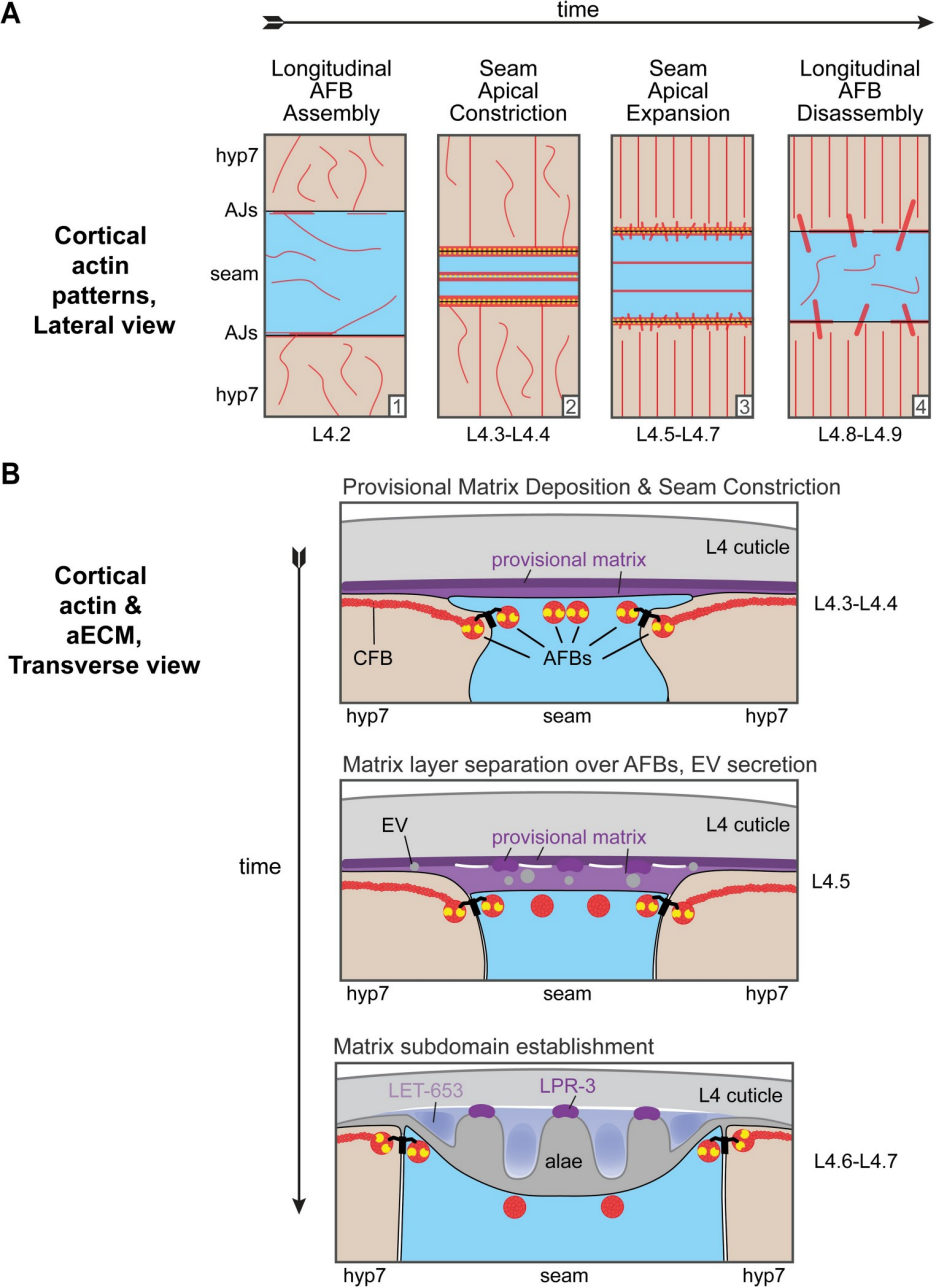
A cytoskeletal relay model for development of the adult alae. Seam is blue, hyp7 peach. Cortical actin networks depicted in red, NM II in yellow, and AJs between seam and hyp7 in black. (A) Graphical representation of cortical actin networks across the larval-to-adult transition. (1) Epidermis prior to longitudinal AFB assembly; (2) Seam narrowing. Longitudinal AFBs (with associated NM II) and poorly aligned hyp7 CFBs are present. The most medial seam AFBs are closely spaced and often unresolvable; (3) Seam widening. Longitudinal AFBs and well-aligned hyp7 CFBs are present. NM II is concentrated at the outer (junctional) AFBs; (4) AFB disassembly and enlargement of transverse actin spikes. (B) Graphical representation of transverse cross-sections through the seam, hyp7, and overlying matrices during early (top), mid (middle), and late (bottom) stages of alae formation. (Early) Seam narrowing and deposition of provisional matrix layers (purples). (Mid) Matrix layer separation over AFBs and beginnings of matrix patterning. Both the seam and hyp7 syncytia secrete EVs. (Late) As alae take shape, distinct provisional matrix components (purples) and cuticle components (grey) accumulate within different subdomains. LPR-3 and collagens concentrate at ridges, LET-653 (and later FBN-1) at valleys, and NOAH-1 at both ridges and valleys.

Actomyosin networks and ZP-containing aECMs both play many roles in shaping epithelial tissues [39,40,58], but how the two systems are coordinated and how the actin cytoskeleton impacts complex aECM shapes remain poorly understood. Here we showed that actomyosin networks are required to pattern a provisional ZP-protein-rich matrix that determines the final structure of acellular alae ridges in the adult cuticle of *C*. *elegans*. Fig 11 summarizes our proposed model for how cytoskeletal patterns are translated into aECM patterns. The four key aspects of this model are: 1) Transient narrowing and then gradual widening of the seam syncytium along the dorsal-ventral axis, through an atypical apical constriction mechanism involving NMII and longitudinal AFBs; 2) Transmission of longitudinal AFB patterns across the seam apical membrane to a newly deposited, ZP-rich, provisional matrix, thereby generating local regions of separation between matrix layers; 3) Concurrent AFB-dependent patterning of provisional matrix components into longitudinal bands that pre-configure the final alae or their flanking valleys; 4) Recruitment of collagens or other cuticle components to provisional matrix bands, thereby propagating regional differences in the short-lived provisional matrix to permanent differences in cuticle matrix structure. Below we discuss each of these aspects in more detail.

### An atypical apical constriction mechanism drives transient narrowing of the seam syncytium

Seam narrowing precedes alae formation, but we found no evidence that forces associated with seam narrowing buckle the apical membrane to pattern ridge-like structures. In all ten TEM specimens examined, including those with very small, nascent alae, the seam plasma membrane appears smooth. Furthermore, we found that alae form as the seam is widening, rather than during the narrowing phase. The purpose of seam narrowing remains unclear, but one advantage could be that it allows hyp7 to secrete matrix components to the narrow region where alae will form.

Seam narrowing appears to occur via an atypical apical constriction mechanism. We showed that seam narrowing does depend on NM II, yet AFBs are oriented in parallel to the direction of tissue shortening rather than perpendicular to it as one would usually expect. Notably, the organization of AFBs in the seam resembles that of AFBs within zebrafish microridges, which are maze-like cellular protrusions on the epidermal surface of mucosal epithelia [59,60]. In that case, NM II-containing minifilaments appear to connect parallel AFBs to generate constrictive forces that pull those AFBs closer together [61]. The spacing between parallel AFBs (500nm-1 micron) is similar in developing zebrafish microridges and *C*. *elegans* seam syncitia, and in the future it will be interesting to test if a similar minifilament-based constriction mechanism could be operating during seam narrowing.

### Differential separation vs. adhesion of provisional matrix layers initiates alae formation

The provisional matrix is a temporary enclosure composed of several ZP-domain proteins (LET-653, NOAH-1, FBN-1), putative lipid transporters (LPR-3), and other still unknown matrix components that infiltrate beneath the cuticle. Many components of the provisional matrix are deposited on the apical surface of both the seam and hyp7 syncytia prior to and during the period of seam narrowing and then subsequently organize into longitudinal bands corresponding to nascent ridges or valleys (Fig 11B). Both ridge- and valley-localized components are needed to shape the final alae. Importantly, none of the matrix factors studied here are unique to the seam, yet they organize into alae-like structures only in this location.

Ultrastructurally, the first sign of matrix band patterning is the appearance of four evenly spaced, small separations between provisional matrix layers (Fig 11B). The position of these initial matrix separations closely matches those of the four underlying seam AFBs present during seam widening. These separations will eventually define the valleys surrounding the adult alae, while the three intervening areas of remaining matrix adhesion define the adult alae ridges themselves. In the absence of LET-653, alae ridges *vs*. valleys appear poorly demarcated, consistent with a key role for the provisional matrix in subregion establishment.

Differential separation vs. adhesion of matrix layers is a novel mechanism for patterning ECM structures, but it could be broadly applicable given the well-known propensity of apical matrices to be organized into distinct layers.

### Relay of the seam AFB pattern to the provisional matrix

We showed that actin assembles into four longitudinal AFBs prior to detectable matrix patterning, and that actin is required to properly pattern the provisional matrix and later cuticle into longitudinal bands that are either offset or aligned with these AFBs (Fig 11B). Therefore, actin acts "upstream" of the matrix factors. While we can’t exclude a purely permissive role for actin, we favor an instructive role because of the striking relationship between the longitudinal AFBs and the observed matrix patterns and ultrastructural separations.

How might AFB patterns be transmitted to the aECM? One possibility is that AFBs provide a downward pulling force that leads to separations in the matrix layers directly above them. This model requires that AFBs and the provisional matrix be somehow connected across the apical plasma membrane. Both NOAH-1 and FBN-1 contain predicted transmembrane domains (Fig 7A) that could link the provisional matrix to the seam apical membrane, but any direct connections to the cytoskeleton are unknown. In hyp7 at later stages, the actin cytoskeleton connects to cuticle aECM via matrilin-related proteins [62,63]. Distinct but analogous linkers may connect the actin cytoskeleton to the provisional aECM over the seam. This mechanical connection model does not need to invoke actomyosin contractility of the seam AFBs to explain the pulling mechanism; rather, as long as a physical connection is present, the observed downward sinking of the seam cell or the continuous motion of the worm’s body could generate sufficient tugging forces on the matrix overlying the AFBs.

A second, not mutually exclusive, possibility is that AFBs direct secretion of matrix proteases or other factors that modify the matrix at specific overlying sites. In Drosophila, NM II-mediated actomyosin contraction facilitates docking and compaction of large vesicles that deliver several aECM components to cell surfaces [64], and cortical actin filaments are thought to specify sites of chitin synthase accumulation for chitin extrusion [65]. Longitudinal AFBs could serve a similar secretory role in the seam. However, as yet we have not observed any evidence for patterned deposition of seam matrix factors or for patterned release of the EVs seen by TEM.

A key feature of the above models is that initially unpatterned matrix factors organize into longitudinal bands in response to an initiating cue from seam AFBs. Once the process is set in motion, self-organizing properties of the matrix may re-enforce the initial differences. For example, we propose that intrinsic biophysical properties could cause each protein to segregate preferentially toward or away from regions of tension, or to adhere preferentially to partners in different (now separated) layers, thereby establishing alternating matrix subregions with different molecular contents (Fig 11B).

### The provisional matrix as a scaffold for permanent matrix assembly

Once the provisional matrix pattern is established, the next challenge is to grow and shape the alae ridges, while gradually replacing the provisional matrix with more permanent cuticle components such as collagens. *C*. *elegans* expresses more than 170 predicted cuticle collagen genes [14], but so far very little is known about the collagen (or other matrix) content of adult alae. The cuticulins important for L1 or dauer alae formation do not seem to play a role in adults [13]. We hypothesize that binding interactions between specific provisional matrix factors and specific permanent matrix factors ultimately recruit different sets of factors to the alae ridges vs. valleys, thereby propagating the provisional matrix patterns to the permanent matrix.

Our TEM data suggest that alae enlargement is preceded by a burst of exocytosis that fills the developing matrix region over the seam with EVs of varying size. Seam EVs were previously suggested to release hedgehog-related cargos [66], and we hypothesize that EVs could also be a major route by which other types of matrix cargo or proteases are delivered to the alae. Many of the EVs appear to derive from hyp7, so (as proposed above) one role of seam narrowing could be to facilitate dumping of contents from hyp7 into a narrow extracellular space over the seam. Some of the larger EVs resemble migrasomes [57] and might simply be debris left behind by hyp7 as it begins to withdraw during seam widening. Once more permanent alae components are identified, it will be interesting to test if they are trafficked though EVs and to investigate how the processes of matrix delivery, infiltration, and replacement occur.

A key conclusion from this study is that provisional matrix proteins can play critical roles in shaping a permanent aECM structure even when these proteins are not present in that final structure. Provisional matrices likely are important intermediates in forming and shaping other permanent aECM structures too. For example, in Drosophila, a temporary luminal matrix precedes formation of a distinct chitin-rich cuticle within tracheal tubes [12]. A dramatic example of a provisional matrix in mammals is that preceding tooth enamel, which initially contains many matrix proteins, most of which are removed and replaced by calcium phosphate during the process of mineralization [9]. In cases where the final aECM structure must be rigid enough to retain its shape, it makes sense to pattern a more malleable provisional matrix first and then to use that matrix as a scaffold or mold on which to build the permanent structure.

## Materials and methods

### Strains and transgenes

*C*. *elegans* strains used in this study are listed in S1 Table. Strains were maintained under standard conditions [67] and cultivated at either 20°C or 25°C, as indicated in Figure legends. For synchronization, gravid worms were allowed to lay eggs during limited time windows or were bleached to isolate eggs and hatchlings arrested in starvation-induced L1 diapause and were released from diapause by plating on NGM seeded with *E*. *coli* OP50-1. The precise stages of L4 worms were further determined based on the shape of the vulva [31,53].

Strains with conditional *nmy-2* alleles were propagated at permissive temperature (15°C) and cultivated at restrictive temperature (25°C) following release from starvation-induced L1 diapause. *let-653* mutant defects were examined in animals rescued to viability with a transgene driven by the excretory duct-specific promoter *lin-48* [68]. *lpr-3* seam mosaics were obtained from transgenic strain UP3452 *[lpr-3(cs231); csEx436 [lpr-3+; myo-2*::*mCherry]*; potential mosaics were recognized based on loss of the transgene-associated *myo-2*::*mCherry* signal in one or more of the three pharyngeal muscle 3 (pm3) cells, two of which derive from the ABa lineage and thus are lineally-related to seam cells [17]. 10/15 such mosaics had alae defects, compared to only 1/21 non-mosaic siblings (p = 0.0001, Fisher’s Exact Test).

S2 Table describes the oligonucleotides used in this study. Phusion High Fidelity Polymerase (NEB) was used to amplify DNA for sequencing and cloning. Gibson assembly (NEB) and standard cloning reactions were used to construct fusion genes and corresponding plasmids. To create the *egl-18p*::*rde-1+ egl-18p*::*rde-1*::*sl2*::*mCherry*::*unc-54* 3′UTR fusion gene housed in pSK08, the promoter of *egl-18*, which corresponds to nucleotides 1910072–1913471 of chromosome IV (GenBank: NC_003282); the coding region of *rde-1*, which corresponds to nucleotides 9988043–9991614 of chromosome V (GenBank: NC_003283); coding sequence for *mCherry* (GenBank: KT175701), *sl2* (GenBank: LK928133); and the *unc-54* 3′UTR cassette from pPD95.75 were combined. To construct the *dpy-7p*::*rde-1+ dpy-7p*::*rde-1*::*sl2*::*nls-gfp*::*unc-54* 3′-UTR fusion gene housed in pSK38, the minimal promoter of *dpy-7*, which corresponds to nucleotides 7537789–7537869 and 7537914–7538219 of chromosome X (GenBank: NC_003284); the coding region of *rde-1*; *SL2*; and the *nls-gfp*::*unc-54* 3′UTR cassette from pPD95.73 were united. To construct the *dpy-7p*::*utrnch*::*dsRed*::*unc-54* 3′UTR fusion gene housed in pSK26, the promoter of *dpy-7* (as above); the coding sequence for the first CH domain (residues 1–261) of human Utrophin (GenBank: LX69086); the coding sequence for *dsRed* (GenBank: HQ418395); the *unc-54* 3′ UTR cassette from pPD95.81; and the pUC57 backbone were combined. To construct the *egl-18p*::*utrnch*::*gfp*::*unc-54* 3′ UTR fusion gene housed in pSK34, the promoter of *egl-18*; the sequence encoding UTRNCH; and the *gfp*::*unc-54* 3′ UTR cassette and backbone from pPD95.81 were united. All variants of plasmid pPD95 were gifts from Andy Fire.

To construct the *noah-1*::*sfGFP*::*noah-1* translational fusion gene housed in pCM05, regulatory and coding regions of *noah-1* were amplified from genomic DNA (nucleotides 5874389–5883950 of chromosome I, GenBank: LK927608) and cloned into a NotI-filled derivate of pCR-Blunt II-TOPO (Invitrogen). A *NotI-sfgfp-NotI* cassette was inserted in-frame between the codons for P624 and V625 of *noah-1a* (Genbank: NM_170870). The corresponding NotI site was created using a Q5 mutagenesis kit (Invitrogen). Superfolder (sf) GFP was isolated from pCW11 (Max Heiman, Harvard University).

All extrachromosomal arrays were generated by microinjection of young adults with mixtures containing 100ng/μl DNA. To generate *aaaEx37*, pSK08 (5ng/μl), *ttx-3*::*gfp* (40ng/μl), and pRS316 (55ng/μl) were co-injected into JK537 *rde-1(ne219)*. To generate *aaaEx162*, pSK38 (5ng/μl), *ttx-3*::*dsred* (40ng/μl) and pRS316 were co-injected into JK537. To generate aaaEx108, pSK26 (0.5ng/μl), *ttx-3*::*gfp*, and pRS316 were co-injected into N2. To generate *aaaEx117*, pSK34 (5ng/μl), *ttx-3*::*gfp*, and pRS316 were co-injected into N2. Optimal plasmid concentrations used to generate tandem utrnch arrays were empirically determined by titration. UTRNCH signals were readily detected in the resulting transgenic animals, while phenotypes associated with high levels of UTRN were not observed. To generate *aaaEx78 [fl-fbn-1*::*mCherry*::*fbn-1]*, pSK27 (2.5 ng/μl), the above-mentioned PCR product (1.15 ng/μl), *ttx-3p*::*gfp*, and pRS316 were co-injected into N2. To generate *aaaEx167*, pCM05 (1ng/μl), *ttx-3*::*dsred*, and pRS316 were co-injected into ARF379. Resulting transgenic lines were out-crossed to N2 to remove *aaaIs12*. The extrachromosomal arrays *aaaEx78* and *aaaEx167* rescued lethality caused by respective null alleles of *fbn-1* or *noah-1*, confirming the production of functional fusion proteins. Extrachromosomal arrays were integrated into the genome by UV irradiation at 450 kJ using an FB-UVXL-1000 (Fisher Scientific). Strains with newly integrated arrays were back crossed to JK537 or N2 4 to 6 times prior to further analyses.

### RNA-mediated interference (RNAi)

Bacterial-mediated RNAi was performed as described [69], except that NGM (nematode growth medium) plates were supplemented with 8mM rather than 1mM, isopropyl β-D-1-thiogalactopyranoside (IPTG). For attenuated RNAi treatments, animals were washed off from control plates 14hrs after release from L1 diapause with 14 ml M9, rotated for 30 minutes in M9 to remove residual gut bacteria and then transferred to experimental RNAi plates. As a control, worms were fed the same *E*. *coli* HT115(DE3) transformed with the empty vector pPD129.36. Upon induction by IPTG, such bacteria produce short dsRNA molecules that do not match any annotated gene of *C*. *elegans*.

To knock down *actin* by bacterial-mediated RNAi, we used a sequence-verified clone for *act-2* present in the Ahringer library [69]. To knock down *nmy-1* (Genbank: LK927643), 1121 bp of genomic DNA from exon 10 was cloned into pPD129.36, the standard expression vector for dsRNAs. For *zoo-1* (GenBank: NM_001026515), cDNA spanning exons 1–7 was cloned into pPD129.36, as previously described [44]. For *noah-1* (GenBank: LK927608), 1024bp from exon 6 was cloned into pPD129.36. Each of the resulting plasmids (pSK43, pSK44 and pCM13) was verified by Sanger sequencing and used to transform *E*. *coli* strain HT115(DE3).

### DiI staining of cuticles

DiI staining to visualize cuticle structures was performed essentially as described [32]. Briefly, approximately 600 adult worms were incubated in 400 μl of 30 μg/mL DiI (Sigma) in M9 for 3 hours, shaking at 350 rpm. Worms were then washed 1X in M9 buffer, re-suspended in 100 μl of M9, and dispensed to a 6-cm NGM plate seeded with *E*. *coli* OP50-1. To remove excess unbound dye, worms were allowed to crawl on the plate for 30 minutes prior to imaging.

### Light microscopy and image analyses

Worms were anesthetized with sodium azide (2.5%) and/or levamisole (10 mM) in M9 buffer and mounted on 2% agarose pads. A Zeiss Axioplan or Axioskop microscope (Carl Zeiss Microscopy) with an attached Hamamatsu Orca ER CCD camera or Leica DFC360 FX camera was used for compound microscopy. Images were acquired and analyzed using the software packages Volocity 6.3 (PerkinElmer) or Qcapture (Qimaging). The confocal images in Figs 2 and 4 and 5C, were captured on a Zeiss LSM5 controlled by ZEN 9.0 software. Images in Figs 3 and 8B, and the NOAH-1 and FBN-1 images in Fig 7, were captured on a Zeiss LSM880 with Airyscan processing. Confocal images of LET-653 and LPR-3 in Fig 7B were captured with Leica TCS SP8 confocal microscope. Confocal images in Figs 5A and 5B and 8A and 8C were captured with a Leica DMi8 confocal microscope. Image intensity and color were adjusted in FIJI or Photoshop for presentation. Figure legends or graphs indicate the number of specimens imaged for each experiment, but at minimum, all images are representative of at least n = 5 per marker per stage.

Alae defects were quantified using a scoring rubric that prioritized regions of disorganized alae over gaps where alae were completely missing; animals that displayed each phenotype in different body regions were placed in the former category. Measurements were made using Volocity 6.3 (PerkinElmer), ImageJ (Version 1.48v, NIH), and Fiji [70]. Seam width was measured in Fiji as the distance between AJM-1-marked junctions; six measurements at 100 point intervals were made per image and averaged. For NM II knockdown experiments, where seam shape was too abnormal for standard width measurements, normalized seam width was calculated based on seam area (surface area within AJM-1::mCHERRY boundaries, measured in Volocity), divided by the distance imaged along the A-P axis. The ImageJ plugin FibrilTool was used to measure CFB anisotropy [71]. For each worm assayed, 6 values were obtained by subdividing the lateral region of hyp7 into 3 dorsal and 3 ventral ROIs, each approximately 400 μm^2^.

### Transmission electron microscopy

For TEM in Fig 1, wild-type (N2) young adults were collected, washed once in 8% ethanol and M9, and washed 3 times in PBS over a 30-min period. Specimens were suspended in 2.5% glutaraldehyde, 1% paraformaldehyde, and 0.1M sucrose in PBS; incubated for 2 hours on ice; and incubated for 16 hours at 4°C. Samples were washed, post-fixed in 1% OsO4, and dehydrated by serial immersion in graded ethanol solutions. Samples were then passed through propylene oxide, embedded in serial steps using mixtures of propylene oxide and Epon 812, and cured at 60°C for 48 hrs. An RMC MTX ultramicrotome was used to cut 60 nm sections, which were stained with uranyl acetate and lead citrate. Sections were observed using a 100CX JEOL electron microscope (Jeol, Peabody Massachusetts).

For TEM in Figs 9 and 10 and S1, synchronized wild-type (N2) and *let-653* mutant (UP3342) L4 animals were collected and processed by high pressure freezing followed by freeze substitution into osmium tetroxide in acetone [54,55]. Specimens were rinsed and embedded into LX112 resin and cut transversely to generate thin sections of approximately 70 nm each. At least two sections each from two different mid-body regions were sampled per animal. Sections were stained with uranyl acetate and lead citrate and observed on a JEM-1010 or JEOL1400Plus (Jeol, Peabody Massachusetts) transmission electron microscope. Images were processed in ImageJ and manually pseudocolored in Adobe Illustrator (Adobe, San Jose California). We imaged a total of n = 10 N2 and n = 2 UP3342 specimens.

### Statistical analyses

GraphPad Prism and Microsoft Excel were used for statistical analyses. In all dot-plots or bar graphs, lines and error bars indicate the mean and standard deviation, and dots represent mean measurements from individual animals. To perform statistical analyses on categorical data, phenotypic categories were combined such that outcomes were classified as abnormal versus superficially normal (Figs 2 and 6). Numerical data used for all graphs are provided in Supporting Information files S1–S8 Data.

## Supporting information

S1 Fig Cortical actin and provisional matrix patterns during L3 and early L4. A) Single confocal slices showing cortical actin (Pseam::UTRNCH::GFP, green) and apical junctions (AJM-1::mCHERRY, magenta) in animals at the indicated stages (strain ARF404, 25°C). Although some longitudinal actin bundles were present during L3, much of the cortical actin oriented transversely (n = 6). After seam cell division, most cortical actin oriented longitudinally (n = 7). B) Provisional matrix components appeared diffuse and unpatterned over the L3 seam during synthesis of the L4 cuticle (which lacks alae). Left, strain UP3666, 20° C. Right, strain CM10, 20° C. Animals were staged based on the division status of the Pn.p vulva precursor cells. Images are single confocal slices and representative of at least n = 5 animals per stage.
(PDF)

S2 Fig Outlier wild-type L4 TEM specimen with broken and buckled cuticle. Cuticle breaks may release mechanical tension and allow buckling of matrix over the seam. Seam is false colored in blue. Boxes indicate regions shown in panels to right. A, A’) N2 TEM section through a mid-body region far from the vulva. The cuticle over the seam is arranged into deep folds. We interpret this cuticle to be that of the mid-L4 stage. Note mature appearance of the cuticle, small seam cell size, and absence of receding cuticle or lamellar LROs typically associated with late L4s (compare to sibling specimens in Figs 9 and 10). B, B’) TEM section through the vulva region of the same specimen. A large break in the L4 cuticle is present at the vulva, which has a large lumen characteristic of mid-L4s and no signs yet of adult cuticle formation. Note connection of dorsal vulva cells to the seam and distortion of seam cell shape in this region. Scale bars: 5 μm.
(PDF)

S1 Table C. elegans strains used in this study. (XLSX)

S2 Table Oligonucleotides used in this study. (XLSX)

S1 Data Source data for Fig 2D graph. (XLSX)

S2 Data Source data for Fig 2F graph. (XLSX)

S3 Data Source data for Fig 3C graph. (XLSX)

S4 Data Source data for Fig 4D graph. (XLSX)

S5 Data Source data for Fig 5D graph. (XLSX)

S6 Data Source data for Fig 5E graph. (XLSX)

S7 Data Source data for Fig 6B graph. (XLSX)

S8 Data Source data for Fig 6D graph. (XLSX)
