# Supplementary figures and images for: A transient apical extracellular matrix relays cytoskeletal patterns to shape permanent acellular ridges on the surface of adult C. elegans
Source: PLoS Genet. 2022 Aug 12;18(8):e1010348. doi: 10.1371/journal.pgen.1010348 (PMC9401183; doi:10.1371/journal.pgen.1010348)

**A****F-actin + Junctions**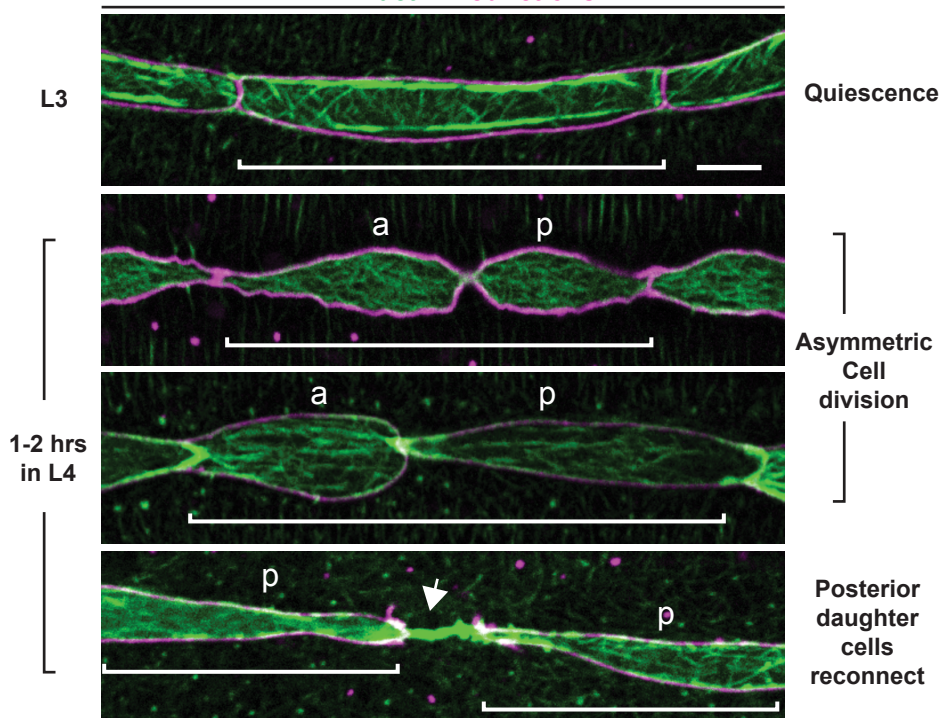**B****SfGFP::LPR-3****NOAH-1::SfGFP**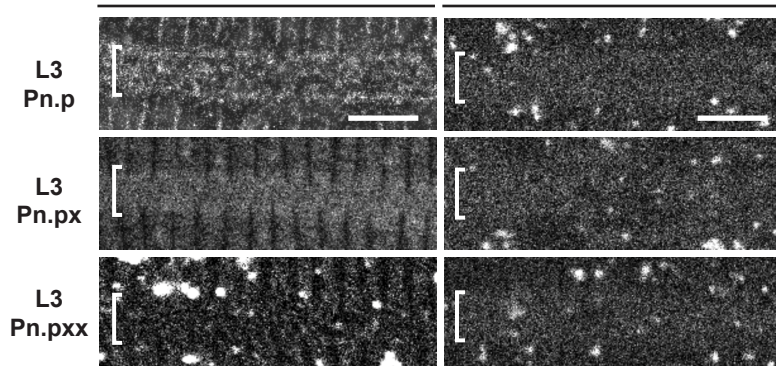

Supplement: S1 Fig — A) Single confocal slices showing cortical actin (Pseam::UTRNCH::GFP, green) and apical junctions (AJM-1::mCHERRY, magenta) in animals at the indicated stages (strain ARF404, 25°C). Although some longitudinal actin bundles were present during L3, much of the cortical actin oriented transversely (n = 6). After seam cell division, most cortical actin oriented longitudinally (n = 7). B) Provisional matrix components appeared diffuse and unpatterned over the L3 seam during synthesis of the L4 cuticle (which lacks alae). Left, strain UP3666, 20° C. Right, strain CM10, 20° C. Animals were staged based on the division status of the Pn.p vulva precursor cells. Images are single confocal slices and representative of at least n = 5 animals per stage. (PDF) [file pgen.1010348.s001.pdf]

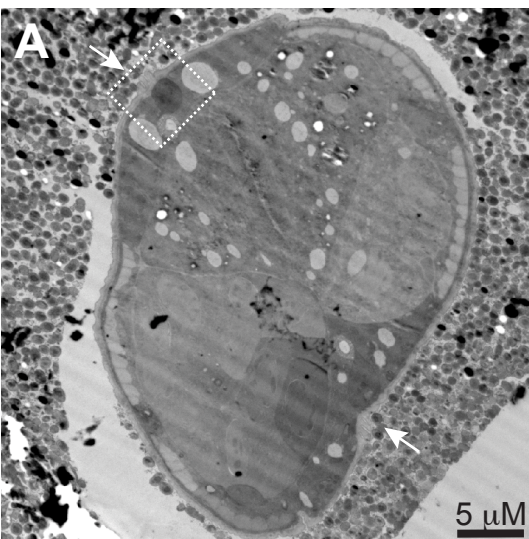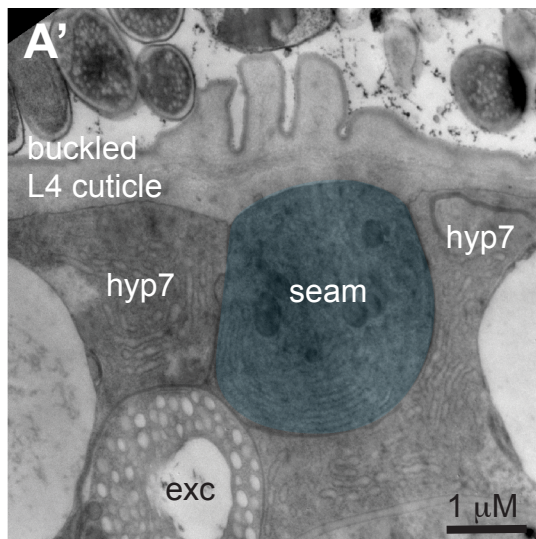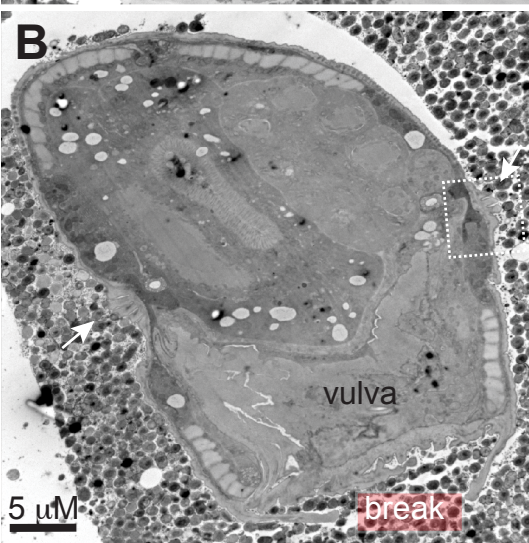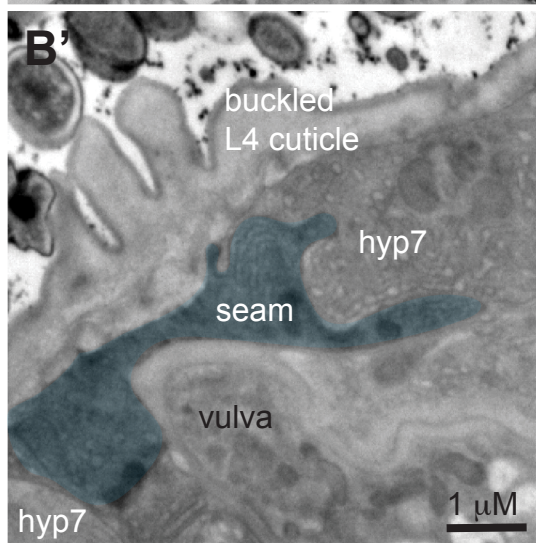

Supplement: S2 Fig — Cuticle breaks may release mechanical tension and allow buckling of matrix over the seam. Seam is false colored in blue. Boxes indicate regions shown in panels to right. A, A’) N2 TEM section through a mid-body region far from the vulva. The cuticle over the seam is arranged into deep folds. We interpret this cuticle to be that of the mid-L4 stage. Note mature appearance of the cuticle, small seam cell size, and absence of receding cuticle or lamellar LROs typically associated with late L4s (compare to sibling specimens in Figs 9 and 10). B, B’) TEM section through the vulva region of the same specimen. A large break in the L4 cuticle is present at the vulva, which has a large lumen characteristic of mid-L4s and no signs yet of adult cuticle formation. Note connection of dorsal vulva cells to the seam and distortion of seam cell shape in this region. Scale bars: 5 μm. (PDF) [file pgen.1010348.s002.pdf]
